# Visible-light–triggered BMP-2 release from enzymatically crosslinked marine collagen–alginate hydrogel blends enhances osteogenesis in dental pulp stem cells

**DOI:** 10.3389/fphys.2026.1743209

**Published:** 2026-02-17

**Authors:** Francesco Torelli

**Affiliations:** 1 SOSD Odontostomatologia Chirurgica e Speciale, Ospedale Riuniti delle Marche, Ancona, Italy; 2 Microfluidic Innovation Centre, Paris, France

**Keywords:** alginate porogen, BMP-2, coumarin photocage, dental pulp stem cells, enzymatic crosslinking, light-triggered release, marine collagen, osteogenesis

## Abstract

**Objectives:**

Achieving spatiotemporal control over osteoinductive signaling remains a key challenge in craniofacial tissue engineering. Conventional BMP-2 delivery from photocrosslinked hydrogels often leads to uncontrolled burst release and cytotoxic by-products from radical initiators. Here, we designed an enzymatically crosslinked marine collagen–alginate hydrogel blend that enables visible-light–triggered, on-demand release of BMP-2 while promoting oxygen diffusion through leachable porosity.

**Methods:**

Marine collagen functionalized with thiol groups (collagen-SH) was crosslinked by microbial transglutaminase (mTG) under physiological conditions, avoiding light-initiated polymerization. Recombinant BMP-2 was conjugated via a coumarin-based 405 nm–cleavable linker (BMP-2_pc) and covalently tethered to the collagen network. Non-crosslinked sodium alginate (0.6% w/v) was incorporated as a sacrificial porogen to create micropores upon diffusion. DPSC were encapsulated (1.5 × 10^6^ cells/mL) and subjected to daily blue-LED pulses (405 ± 10 nm, 25 mW cm^−2^, 60 s) for up to 14 days. BMP-2 release (ELISA), porosity (SEM), oxygen diffusivity (Clark microelectrode), viability, and osteogenic differentiation (ALP, qPCR, Alizarin Red) were assessed.

**Results:**

Blue-light stimulation induced stepwise BMP-2 release (≈23% per pulse; 60% cumulative at 72 h), while mTG crosslinking preserved coumarin integrity. Alginate leaching generated an interconnected microporosity (20–60 µm pores) and increased oxygen diffusion coefficient by 42% ± 9%. DPSC viability remained >90%. Light-pulsed composites exhibited 2.4-fold ALP activity and 2.8-fold higher mineral deposition versus dark controls (p < 0.01).

**Conclusion:**

The orthogonally crosslinked marine collagen–alginate composite supports visible-light–controlled BMP-2 delivery and oxygen-enhanced osteogenesis without photoinitiator toxicity. This platform provides a modular, sustainable route toward clinically programmable scaffolds for dental and craniofacial regeneration.

## Introduction

Bone morphogenetic protein 2 (BMP-2) remains the benchmark osteoinductive cue for craniofacial and dental tissue regeneration (Yang et al., 2015; Vantucci et al., 2021; Jeon et al., 2022; Zhang et al., 2018; Qi et al., 2024). Its ability to trigger differentiation of mesenchymal and neural-crest–derived progenitors such as dental pulp stem cells (DPSC) has long been exploited in bone and dentin engineering. Nevertheless, the clinical translation of BMP-2 has been hindered by the limitations of current delivery vehicles. Conventional carriers - typically collagen sponges, polylactide microspheres, or photocrosslinked hydrogels - release the protein in an uncontrolled burst during the first 24 h, followed by a steep decline (Vantucci et al., 2021; Jeon et al., 2022). This kinetic mismatch between supply and cellular demand results in off-target mineralization, local inflammation, and rapid loss of bioactivity (Jeon et al., 2022; Zhang et al., 2018). Achieving spatiotemporal control over growth-factor release therefore remains a major challenge in craniofacial biofabrication (Qi et al., 2024; Yamada et al., 2023).

Light-responsive chemistry offers an elegant solution to this problem. Photocleavable linkers can sequester biomolecules within a matrix and liberate them only upon exposure to light of a chosen wavelength, enabling user-defined dosing without invasive manipulation. Unlike enzymatic or diffusion-driven release, photochemical systems provide immediate, reversible, and quantitative control - effectively transforming the growth-factor reservoir into an “optical switch.” However, most previous photocaged systems rely on ultraviolet (UV) activation, where high photon energies introduce cytotoxicity through DNA damage and reactive oxygen species (Weinstain et al., 2020). The development of visible-light–sensitive photocages, such as coumarin or nitrobenzyl derivatives tuned to 400–420 nm, allows safe activation within the spectral range of standard dental blue-LED devices. These technologies could enable chairside triggering of therapeutic release, bridging laboratory innovation and clinical usability.

Previous work has commonly combined photocages with Gelatin Methacrylate (GelMA), a photocrosslinkable derivative of collagen (Nguyen et al., 2019; Nguyen et al., 2020; Monteiro et al., 2018; Klotz et al., 2016). While GelMA offers tunable mechanics and cell adhesion, its requirement for free-radical polymerization under blue or UV light complicates integration with visible-light photocages: both reactions compete for the same wavelength range. The radical polymerization step can prematurely cleave or deactivate coumarin linkers, undermining the precision of photochemical release. Furthermore, residual photoinitiators such as LAP or Irgacure can introduce cytotoxicity or oxidative stress detrimental to stem-cell differentiation (Nguyen et al., 2019; Nguyen et al., 2020). To decouple matrix formation from photorelease, a non-photochemical crosslinking mechanism is required.

To address this, we selected marine collagen as the base polymer and microbial transglutaminase (mTG) as the enzymatic crosslinker (Monteiro et al., 2018; Klotz et al., 2016; Geahchan et al., 2022). Marine collagen, extracted from fish skin or jellyfish, exhibits high biocompatibility, minimal risk of zoonotic transmission, and a natural alignment with marine-derived regenerative biomaterials (Zhai et al., 2025). When functionalized with thiol groups (collagen-SH), its ε-(γ-glutamyl)-lysine residues become efficient substrates for mTG-mediated crosslinking (Lai et al., 2022; Buscaglia et al., 2022). This enzymatic process proceeds under physiological temperature and pH, requires no photoinitiators, and preserves the integrity of photosensitive moieties (Buscaglia et al., 2022). The resulting network recapitulates the fibrillar ultrastructure of native extracellular matrix while providing tunable stiffness in the 1–5 kPa range - ideal for DPSC encapsulation and early osteogenic induction. Importantly, decoupling polymerization from illumination allows the visible-light step to be dedicated exclusively to the photo-triggered BMP-2 release, ensuring clean, orthogonal control over both processes.

A second obstacle in hydrogel-based bone regeneration is oxygen diffusion. Densely crosslinked matrices hinder gas transport, producing hypoxic cores that limit cell proliferation and mineral deposition (Annabi et al., 2010; Jiang et al., 2024). Strategies such as microchannel printing or oxygen-releasing particles can mitigate this, but they often require complex fabrication steps or generate reactive by-products. A simpler, inherently biocompatible alternative is to incorporate a sacrificial porogen that transiently occupies space within the forming network and subsequently leaches out, leaving an interconnected microporosity that facilitates nutrient and oxygen diffusion (Saeki et al., 2020; Li et al., 2025).

Sodium alginate provides an ideal candidate for such a sacrificial phase. It is hydrophilic, non-toxic, and easily removed under mild conditions (Annabi et al., 2010; Sahoo and Biswal, 2021). In its non-crosslinked form, alginate behaves as a viscous polysaccharide that can be mixed with the collagen-SH precursor solution and gradually diffuse out after enzymatic gelation. This transient alginate phase acts as a porogen: while the mTG crosslinks the collagen network, alginate chains hinder local compaction and generate voids on the order of tens of micrometres. Once the alginate diffuses into the surrounding medium over 24–48 h, it leaves behind micropores that improve gas exchange and facilitate subsequent extracellular-matrix deposition (Vanlauwe et al., 2024). Compared to rigid microsphere templates, leachable alginate maintains a soft microenvironment conducive to stem-cell encapsulation and avoids abrupt stiffness gradients.

The question arises whether cell encapsulation remains viable in the presence of a leaching porogen. Because mTG crosslinking occurs under physiological conditions and the alginate is water-soluble rather than crosslinked with calcium, DPSC can indeed be suspended within the precursor solution before gelation. The enzymatic network forms around both the cells and the alginate chains; as alginate diffuses out, it gently increases local porosity without exposing cells to osmotic or mechanical stress. Previous studies have demonstrated that enzymatically crosslinked collagen–alginate interpenetrating networks support high cell viability and migration, confirming the feasibility of this approach (Hu and Lo, 2021; Du et al., 2024). Therefore, a marine collagen-SH/mTG hydrogel containing non-crosslinked alginate as a sacrificial porogen provides an effective balance between structural integrity, oxygen permeability, and cytocompatibility.

Within this matrix, BMP-2 can be covalently tethered through a coumarin-based photocleavable linker (Weinstain et al., 2020; Ruskowitz and DeForest, 2018). The conjugation strategy involves coupling BMP-2 to a coumarin-PEG-NHS ester, introducing a 405 nm-sensitive bond between the protein and the collagen backbone (Ruskowitz and DeForest, 2018). Under blue-light exposure, the coumarin moiety undergoes a [2 + 2] cycloreversion, releasing native BMP-2 without residual tether fragments (Azagarsamy and Anseth, 2013). Because the crosslinking chemistry (mTG) is independent of light, the scaffold can be pre-formed and loaded with BMP-2_pc, then activated non-invasively at any chosen time point. This orthogonality between matrix formation and release provides unprecedented control: clinicians or researchers can implant the hydrogel in its inactive state and later trigger growth-factor liberation *in situ* by brief LED illumination.

For dental pulp stem cells, which exhibit strong osteogenic and angiogenic potential (Mortada and Mortada, 2018), such temporal precision could significantly enhance differentiation efficiency. Continuous exposure to BMP-2 often leads to rapid receptor desensitization and aberrant mineralization, whereas pulsatile or short-term signaling more closely mimics developmental dynamics (Vantucci et al., 2021; Zhu et al., 2022). Delivering BMP-2 in controllable bursts may therefore promote orderly matrix maturation and reduce the required protein dose. At the same time, the microporosity introduced by alginate leaching ensures adequate oxygenation, supporting mitochondrial metabolism and preventing hypoxia-induced apoptosis - an essential consideration in thick, diffusion-limited constructs.

The marine origin of the collagen component further reinforces the sustainability and bioethical dimension of the system. Sourcing collagen from marine by-products aligns with circular-economy principles and reduces reliance on mammalian materials, which carry risks of immunogenicity and cultural restriction (Almeida and Vieira, 2025; Dondero et al., 2025). This approach fits within broader approached of marine-derived biodesign, where regenerative materials emulate marine ecology both functionally and symbolically (Santos Filipe et al., 2024).

In summary, this study introduces a visible-light-activated, enzymatically crosslinked marine-collagen hydrogel engineered for precise BMP-2 delivery and improved oxygen diffusion. The design rests on three converging innovations: (1) evaluation of substituting standard photopolymerizable biomaterials with mTG-crosslinked collagen-SH to prevent interference between photocuring and photorelease (Chen et al., 2005); (2) incorporation of non-crosslinked alginate as a sacrificial porogen to generate microporosity and enhance oxygen transport (Saeki et al., 2020); and (3) use of a coumarin photocage enabling blue-light–triggered, on-demand release of bioactive BMP-2 (Ruskowitz and DeForest, 2018; Bojtár et al., 2020).

The specific objectives were therefore:

to synthesize and characterize a 405 nm-cleavable BMP-2 conjugate (BMP-2_pc) covalently anchored to marine collagen-SH;
to integrate non-crosslinked alginate as a leachable porogen and quantify its effects on microarchitecture and oxygen diffusivity;
to assess the viability and osteogenic differentiation of DPSC encapsulated within the composite; and
to benchmark performance against free BMP-2, non-porous collagen hydrogels, and non-irradiated controls.

By combining enzymatic crosslinking, sacrificial porosity, and photo-programmed growth-factor release, the present work seeks to establish a modular platform for light-controlled craniofacial regeneration. The resulting material - rooted in marine biopolymers yet guided by photonic precision - embodies the next-generation of sustainable, adaptive scaffolds capable of synchronizing biological processes with optical command.

## Materials and methods

### Materials

Commercial thiolated gelatin (Gel–SH) derived from cold-water fish skin (15 mol % thiolation, Mw ≈ 100 kDa) was obtained from Rousselot (Gent, Belgium). Microbial transglutaminase (mTG, >100 U g^−1^) was purchased from Ajinomoto Foods, Japan. Recombinant human BMP-2 (carrier-free) and sodium alginate (medium viscosity, 200–300 cP) were supplied by Sigma-Aldrich (St. Louis, MO, United States). The coumarin-PEG-maleimide linker (λ_max 405 nm) was obtained from Iris Biotech (Marktredwitz, Germany). Cell culture reagents (α-MEM, FBS, antibiotics, supplements) were from Gibco/Thermo Fisher. A dental pulp stem cells (DPSC) line was acquired from Lonza. All biomaterial and cell culture work was carried out under sterile biosafety conditions. All aqueous solutions were prepared in sterile phosphate-buffered saline (PBS, pH 7.4).

**FIGURE 1:**
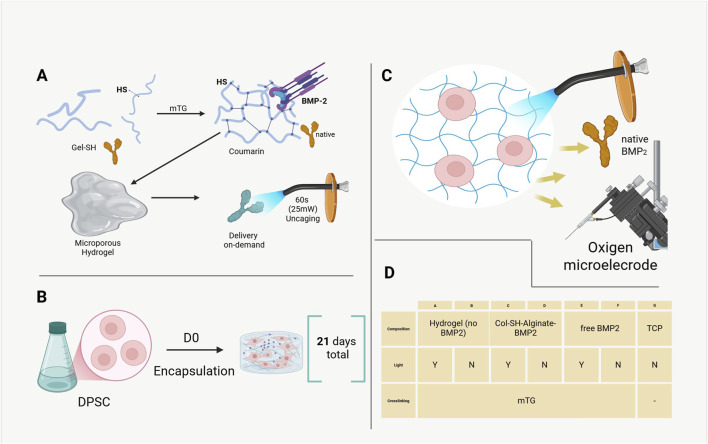
Schematic representation of the visible-light–activated Gel–SH/mTG hydrogel system for on-demand BMP-2 release and dental pulp stem cell (DPSC) osteogenesis. **(A)** Enzymatic crosslinking of thiolated gelatin (Gel–SH) via microbial transglutaminase (mTG), with BMP-2 tethered to the matrix through a coumarin photocleavable linker. A brief 405 ± 10 nm light pulse (25 mW cm^−2^, 60 s) releases native BMP-2 into the surrounding matrix. **(B)** Experimental timeline: DPSC encapsulation (D_0_) followed by daily 60 s blue-light pulses for 21 days. **(C)** Incorporation of alginate microporosity enhances oxygen diffusion within the hydrogel, profiled using a Clark-type microelectrode. **(D)** Summary of experimental groups (A–G).

An overview of the experimental design can be appreciated in Figure 1.

### Synthesis of photocaged BMP-2 conjugate (BMP-2_pc)

Recombinant BMP-2 (0.2 mg mL^−1^ in PBS, pH 7.4) was reacted with a 10-fold molar excess of coumarin-PEG-NHS for 1 h at 4 °C (Song et al., 2009). After quenching with 50 mM ethanolamine (pH 8.0, 10 min), unreacted linker was removed by centrifugal filtration (10 kDa, Amicon Ultra). The resulting BMP-2_pc contained a terminal maleimide group for thiol-specific coupling, leveraging maleimide–thiol chemistry as previously described (Song et al., 2009; Pohl et al., 2013). SDS-PAGE confirmed a mobility shift of ≈5 kDa, and BMP-2 immunoreactivity remained >85% by ELISA (reflecting retained activity after conjugation) (Pohl et al., 2013; Chang et al., 2015).

### Coupling of BMP-2_pc to Gel–SH

Gel–SH was dissolved at 10% w/v in sterile PBS at 37 °C for 30 min. BMP-2_pc was added to achieve 200 ng BMP-2 per 50 µL gel. The mixture was maintained at 37 °C, pH 7.2–7.4 for 30 min to allow maleimide–thiol Michael addition (thiol–maleimide coupling kinetics as previously described (Jansen et al., 2018; Darling et al., 2016; Shamsipur et al., 2023; El-Sherbiny and Yacoub, 2013)). This covalent tethering prevented premature protein diffusion prior to photo-cleavage. No initiator or light was used. Furthermore, to confirm photocleavage efficiency and kinetics independently of the hydrogel, BMP-2_pc was also diluted in PBS (0.1% BSA) to the working concentration used in gels and placed in low-protein-binding wells (n = 3). Samples were irradiated at 25 mW cm^−2^, 405 nm for 0, 10, 20, 40, 60, 120, 180, 300 s. Immediately after each exposure, aliquots were withdrawn, chilled on ice, and centrifuged (10 kDa MWCO, 4 °C, 10 min) to remove linker fragments. Released (“free”) BMP-2 was quantified by ELISA (R&D Systems) using a standard curve prepared in matched buffer. Data were fit to a mono-exponential model to estimate apparent cleavage half-life (t½,app) and plateau. Control samples kept in the dark across the same timepoints defined baseline signal (Supplementary Figure S1).

### Preparation of sacrificial alginate porogen

To enhance oxygen and nutrient transport, non-crosslinked alginate inclusions were incorporated as sacrificial porogens (El-Sherbiny and Yacoub, 2013). A 2% w/v sterile sodium alginate solution was prepared in deionized water and filtered (0.22 µm) (Tomić et al., 2023). Immediately before gelation, Gel–SH/BMP-2_pc solution and alginate were combined 4:1 (v/v) using a dual-syringe static mixer to generate transient droplets (≈20–150 µm) within the prepolymer.

### Hydrogel formation and enzymatic crosslinking

Microbial transglutaminase (mTG) was freshly prepared at 15 U g^−1^ gelatin and added to the Gel–SH/alginate blend to initiate crosslinking via ε-(γ-glutamyl)-lysine bonds (Yang et al., 2016; Gupta et al., 2021). For cell-free formulations, the reaction proceeded 45 min at 37 °C in PDMS molds (6 mm × 1.8 mm) (Yang et al., 2016). For cell-laden gels, mTG was diluted in α-MEM and filtered (0.22 µm) immediately before use to maintain sterility.

### Illumination system and LED calibration

A collimated 405 ± 10 nm LED (Thorlabs M405L4, collimation package) was used for photoactivation. Irradiance was calibrated before each experiment by placing a calibrated photoradiometer sensor (e.g., Thorlabs S120VC with PM100D) flush with the gel surface in the identical culture setup (medium volume, vessel, working distance) - consistent with protocols for *in vitro* LED irradiation calibration (Hopkins et al., 2016). Drive current was adjusted to achieve 25 mW/cm^2^ at the sample center. Uniformity across the illuminated area was verified at a 3 × 3 grid (center and 8 compass points, 5 mm spacing); experiments proceeded only if spatial variation was ≤ ±5% of the center value. Exposure time was controlled electronically (60 s), and surface temperature rise was monitored by infrared microprobe (to exclude thermal artifact) (Zein et al., 2018). LED output stability was checked at the start and end of each session (Supplementary Figure S2).

### Physicochemical and optical characterization

Dynamic oscillatory tests were performed on an Anton Paar MCR 302 rheometer (8-mm plate, 37 °C, 1% strain, 1 Hz). Mean storage (G′) and loss (G″) moduli were obtained from n = 3 - consistent with standard small-amplitude oscillatory shear protocols for hydrogels and as indicated by the manufacturer. Swelling ratios were calculated as (W_s_–W_d_)/W_d_, where W_d_ is the dry weight after lyophilization, and W_s_ the weight after 24 h PBS swelling (Park et al., 2009; Feng and Wang, 2023). UV–Vis spectra (350–700 nm; Shimadzu UV-2600) confirmed >60% transmission at 405 nm through 1.8 mm gels - a method analogous to prior hydrogel optical transmission studies (Sornkamnerd et al., 2017).

### hDPSC expansion and encapsulation

Cryopreserved DPSC were expanded in α-MEM +10% FBS +1% penicillin/streptomycin +2 mM L-glutamine at 37 °C and 5% CO_2_, using culture conditions validated for human dental pulp stem cells (Gronthos et al., 2000; Yamada et al., 2025) Cells were detached (TrypLE Express), resuspended in culture medium, and gently mixed with the warm prepolymer at a final density of 1.5 × 10^6^ cells mL^−1^. Fifty-microlitre aliquots were cast into sterile PDMS molds and covered to prevent evaporation. Gelation was completed at 37 °C for 45 min, producing transparent, cell-laden disks. All manipulations were performed under sterile laminar flow. Post-gelation, samples were rinsed three times in warm PBS (5 min each) to dissolve and leach alginate porogens (Wang et al., 2024), yielding interconnected micropores while preserving gel integrity. Constructs were then incubated overnight in complete medium before experimental treatments.

### Experimental group

A synoptic schematic of the experimental and negative and positive control groups can be seen in Table 1. Each group was prepared in, at least, triplicate per assay and timepoint. As it can further be seen in the table, to benchmark the efficacy of the system in selected assay namely differentiation and mineralization ones–see below) against conventional soluble delivery, two TCP positive control groups were added: (H) TCP + soluble BMP-2 (continuous), where BMP-2 was present at every medium change (every 48 h); and (I) TCP + soluble BMP-2 (pulsed), where BMP-2 was applied once per day for 60 min followed by PBS wash and replacement with BMP-2–free osteogenic medium. A conservative dose of BMP2 of 50 ng/mL was selected.

**TABLE 1:** Experimental and control groups.

Group	Composition	Light treatment (405 nm)	Crosslinker
A	Gel-SH only (no BMP-2)	Yes	mTG
B	Gel-SH only (no BMP-2)	No	
C	Gel–SH + BMP-2_pc	Yes	
D	Gel–SH + BMP-2_pc	No	
E	Gel–SH + free BMP-2 (dose-matched, non-caged)	Yes	
F	Gel–SH + free BMP-2 (dose-matched, non-caged)	No	
G	TCP monolayer control	No	-
H	TCP + BMP2 (continuous) positive control	No	-
I	TCP + BMP2 (time-dosed) positive control	No	-

### Photo-triggered BMP-2 release

Photoactivation was performed after encapsulation using a the previously calibrated, and collimated 405 ± 10 nm LED at 25 mW cm^−2^ for 60 s once per day, beginning 24 h after gel formation. Temperature at the gel surface was monitored by infrared probe and remained <1 °C above baseline. For release kinetics, cell-free gels were incubated in 1 mL PBS at 37 °C. Supernatants were collected at 0, 2, 6, 24, 48, and 72 h post-irradiation, frozen at −20 °C, and analyzed by BMP-2 ELISA (R&D Systems). Total immobilized BMP-2 was quantified by exhaustive photolysis (405 nm, 5 min) followed by trypsin digestion (1 μg mL^−1^, 2 h, 37 °C). The stability of the BMP-2–coumarin conjugate under storage and physiological conditions was evaluated by incubating samples in the dark at 4 °C (storage condition) and at 37 °C in PBS (pH 7.4) for up to 24 h. At defined time points (0, 6, and 24 h), samples were subjected to centrifugal filtration (10 kDa MWCO) to separate any prematurely released BMP-2 from the conjugated fraction. Free BMP-2 in the filtrate was then quantified by ELISA (R&D Systems) using matched buffer standards. Also, BMP-2 loading efficiency (LE) was determined by quantifying the total amount of BMP-2 immobilized within the hydrogel network as the ratio between immobilized BMP-2 and the actual BMP-2 mass added to the gel:

$$\begin{array}{ll} \text{LE }\left(\%\right) & =\left(\text{BMP}‐2\text{ initially added}/\text{BMP}‐2\text{ effectively immobilized in the hydrogel}\right)\\ & \;\times 100 \end{array}$$

Cumulative release (%) was calculated as released BMP-2/total × 100. Baseline passive release before the first light exposure was consistently <5% (Francis and DeForest, 2023; Elchiev et al., 2023). An overview of these parameters is shown in Table 2.

**TABLE 2:** Working concentrations and loading efficiency (%).

Sample ID	[BMP2] (ng/mL)	Volume added (mL)	BMP2 recovered (ng)	Photolysis plateau fraction	[*Actual* BMP2]	Corrected immobilized BMP2 (ng)	LE%
Gel 1	3,900	0	174	0.9	195	193.33	99.15
Gel 2	4,000		178		200	198	99
Gel 3	3,850		169		192.5	187.78	97.55

### Oxigen microprofiling

To evaluate oxygen diffusion and reoxygenation dynamics within the hydrogels, dissolved O_2_ microgradients were quantified using a Clark-type microsensor (Unisense OX-50, tip diameter 50 µm) connected to a Unisense PA2000 picoammeter and mounted on a motorized micromanipulator (MM33, Unisense, Aarhus, Denmark). Measurements were conducted under sterile conditions in temperature-controlled medium (37 °C, 5% CO_2_ atmosphere). O_2_ tension was recorded every 100 µm from the hydrogel surface down to 1,000 µm in 100 µm increments, maintaining a 5 s stabilization period at each depth. Each profile was acquired in triplicate positions per sample. Two material formulations were compared: Gel–SH alone, and Gel–SH + alginate porogen. At each depth point, corresponding z-stack images were acquired using a confocal microscope (Zeiss LSM 710, 10× NA 0.45 objective) under the same geometric reference frame (Rivera et al., 2019). Constructs were stained with Calcein-AM (2 µM), Ethidium homodimer-1 (4 µM), and Annexin V–AlexaFluor 647 (5 µL per sample) for 30 min at 37 °C to identify live, necrotic, and apoptotic cells. Viability and apoptosis percentages were quantified by 3D segmentation in ImageJ (Fiji) and normalized to cell density per slice. To assess reoxygenation capacity, profiles were measured at 300 µm depth immediately after medium refresh, and O_2_ µM values were recorded continuously for 10 min. Three independent constructs per condition were analyzed.

### Cytocompatibility and viability

On Days 1, 3, and 7, cell-laden hydrogels were incubated with Calcein-AM (2 µM) and Ethidium homodimer-1 (4 µM) for 30 min at 37 °C (Dominijanni et al., 2021). Confocal images (Zeiss LSM 710; Ex/Em 488/568 nm) were used to calculate viability (%) as live cells on (live cells + dead cells) × 100 from ≥5 fields (n = 3). MTT was measured with a microplate reade according to manufacturers instructions. To isolate the biological effects of coumarin photoproducts generated during photoactivation from those of BMP-2 signaling, conditioned-medium experiments were performed using BMP-2–free hydrogels. Gel–SH hydrogels containing the coumarin photocleavable linker but no BMP-2 were prepared and enzymatically crosslinked with microbial transglutaminase (Weinstain et al., 2020; López-Corrales et al., 2023). Hydrogel disks (50 µL) were incubated in complete α-MEM (1 mL per disk) and exposed to 405 ± 10 nm LED illumination at an irradiance of 25 mW·cm^−2^ for 60 s (radiant exposure: 1.5 J·cm^−2^), matching the illumination protocol used before. Control gels were maintained in the dark. Following illumination, gels were incubated at 37 °C for 1 h to allow diffusion of any soluble photoproducts into the surrounding medium. The resulting conditioned media (CM_light and CM_dark) were collected, sterile-filtered (0.22 µm), and immediately applied to fresh dental pulp stem cell (DPSC) cultures. Then, DPSC were seeded in 24-well plates (2 × 10^4^ cells·cm^−2^) and exposed to conditioned media for 24 h. Cell viability was assessed using MTT and quantified. As a positive cytotoxicity control, cells were briefly exposed to 0.1% Triton X-100. Intracellular reactive oxygen species (ROS) generation was evaluated using the DCFDA assay (Eruslanov and Kursmartsev, 2010). After 30 min exposure to conditioned media, cells were incubated with DCFDA (10 μM, 30 min, 37 °C), washed, and fluorescence was quantified using a microplate reader (Ex/Em 485/535 nm). Hydrogen peroxide (H_2_O_2_, 200 μM, 30 min) served as a positive ROS control (Lee et al., 2017). To assess potential genotoxicity, γH2AX immunofluorescence staining was performed following 6 h exposure to conditioned media. Cells were fixed in 4% paraformaldehyde, permeabilized (0.1% Triton X-100), blocked (3% BSA), and incubated with anti-γH2AX primary antibody (1:500) followed by AlexaFluor-conjugated secondary antibody. Nuclei were counterstained with DAPI. As a positive DNA-damage control, cells were exposed to laminar flow hood UV for 6 h γH2AX-positive nuclei were quantified using ImageJ from ≥300 cells per condition. All experiments were performed using conditioned media generated from at least three independent hydrogel preparations.

### Osteogenic differentiation

After 24 h recovery, constructs were cultured in osteogenic medium (α-MEM +10% FBS +50 μg mL^−1^ ascorbate-2-phosphate +10 mM β-glycerophosphate +10 nM dexamethasone) (Yamada et al., 2025; Khanna-Jain et al., 2012). Medium was changed every 2 days; illumination was maintained daily. On day 7 and 14 lysates (0.1% Triton X-100) were reacted with p-nitrophenyl phosphate substrate; absorbance 405 nm normalized to DNA (PicoGreen) to evaluate ALP activity (Awais et al., 2020). For PCR, Total RNA (TRIzol) was reverse-transcribed (1 µg input, iScript cDNA kit), and amplified (SYBR Green, ABI StepOnePlus) used primers: RUNX2 (F 5′-CCTGAACTCTGCACCAAGTC-3′/R 5′-TGAAACTCTTGCCTCGTCC-3′), ALPL (F 5′-AGGGCAATGAGGTCACATCC-3′/R 5′-GGTGGCAGTGGTGTTGTTGT-3′), OCN (F 5′-CAAAGGTGCAGCCTTTGTG-3′/R 5′-GCGCCTGGGTCTCTTCA-3′). GAPDH served as reference. Relative expression was computed via 2^−ΔΔCt^ normalized to TCP + osteogenic medium. At Day 21, gels were fixed (4% PFA 30 min), stained with Alizarin Red S (40 mM, pH 4.2, 30 min), rinsed, and imaged. Dye was eluted in 10% acetic acid (30 min, 37 °C), and absorbance at 405 nm was measured and normalized to DNA content. For qPCR and Alizarin Red S staining assays TCP benchmark groups were used and processed identically serving as references for continuous and transient BMP-2 exposure. Furthermore, qualitative visualization was performed via Von Kossa (counterstained with nuclear Fast Red) staining. Briefly, hydrogels were rinsed twice in PBS and fixed in 4% paraformaldehyde (30 min, RT). Samples were immersed in 5% (w/v) silver nitrate and exposed to UV light (365 nm, 30 min) until brown–black deposits appeared. Excess silver was removed with 5% sodium thiosulfate (5 min), followed by distilled-water rinses. Constructs were counterstained with nuclear fast red (0.1% in 5% aluminum sulfate, 5 min) to visualize cell nuclei and extracellular matrix. Black deposits were interpreted as phosphate-rich mineralized regions.

### BMP signaling blockade and early signaling analysis

To establish a causal link between light-triggered BMP-2 release and downstream osteogenic signaling, early activation of the canonical BMP pathway was assessed using pharmacological blockade and short-term signaling readouts (Wang et al., 2014; Phimphilai et al., 2006). Gel–SH/mTG hydrogels containing photocaged BMP-2 (BMP-2_pc) were prepared as described above and assigned to the following conditions: (1) BMP-2_pc + Light, (2) BMP-2_pc + Dark, (3) BMP-2_pc + Light + Noggin, (4) Gel–SH only (no BMP) + Light. For BMP pathway inhibition, recombinant human Noggin (R&D Systems) was added to the culture medium at 500 ng·mL^−1^ 60 min prior to illumination and maintained throughout the experiment. This concentration was selected based on established BMP-2 neutralization protocols in mesenchymal stem cells (Ahnfelt-Rønne et al., 2010; Zhang et al., 2024). Hydrogels were exposed to a single 405 ± 10 nm LED pulse (25 mW·cm^−2^, 60 s; radiant exposure 1.5 J·cm^−2^) using the calibrated illumination system described above. Dark controls were handled identically without illumination. At 0 and 2 h post-illumination, constructs were rapidly rinsed in ice-cold PBS and lysed in RIPA buffer supplemented with phosphatase and protease inhibitors. Protein concentration was determined by BCA assay. Equal amounts of protein were subjected to SDS-PAGE and transferred to PVDF membranes. Membranes were probed with antibodies against phosphorylated SMAD1/5/8 (p-SMAD1/5/8), total SMAD1, and β-actin (loading control). Bands were visualized by chemiluminescence and quantified by densitometry. p-SMAD levels were normalized to total SMAD1 and expressed relative to dark controls (Kopf et al., 2012). Furthermore, immunofluorescence was evaluated. Constructs were fixed in 4% paraformaldehyde at 1 h post-illumination, cryoprotected, and sectioned (20–30 µm). Sections were permeabilized and stained with anti-RUNX2 primary antibody followed by AlexaFluor-conjugated secondary antibodies. Nuclei were counterstained with DAPI. Confocal images were acquired using identical acquisition parameters across groups. Nuclear and cytoplasmic RUNX2 intensities were quantified using automated segmentation in Fiji (ImageJ), and results were expressed as nuclear/cytoplasmic (N/C) fluorescence ratios or percentage of cells exhibiting nuclear RUNX2 enrichment.

### Intracellular ROS assay (DCFDA)

hDPSC encapsulated in Gel–SH/mTG hydrogels were incubated with DCFDA (10 μM, 30 min, 37 °C) in phenol-red–free medium, washed, and exposed to 405 nm light (25 mW·cm^−2^, 60 s; radiant exposure 1.5 J·cm^−2^) (Dominijanni et al., 2021; Eruslanov and Kursmartsev, 2010). Fluorescence was acquired immediately (and optionally at 15–120 min) using identical imaging settings for all groups (Hopkins et al., 2016; Zein et al., 2018). A positive control was prepared by treating cells with H_2_O_2_ (200 μM, 25 min) prior to imaging (Eruslanov and Kursmartsev, 2010). Quantification was performed in Fiji by measuring mean DCFDA intensity per field and normalizing to nuclei count (DAPI). Data are mean ± SD (n = 3 independent constructs; 5 fields per construct).

### Statistical analysis

All experiments were performed in triplicate or quadruplicate independent runs. Results are expressed as mean ± SD. Data normality was assessed using the Shapiro–Wilk test. Differences between formulations and illumination conditions were evaluated by two-way ANOVA followed by Sidak *post hoc* comparisons (α = 0.05). Statistical analysis and plots were generated with GraphPad Prism 10.

## Results

### Rheology, optical and structural stability

**FIGURE 2:**
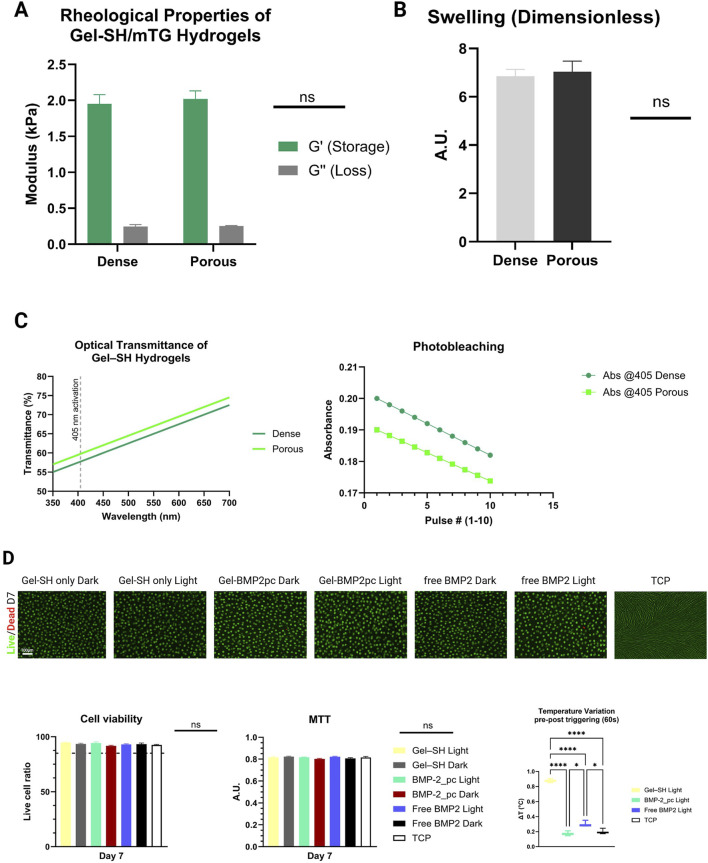
Physicochemical stability and cytocompatibility of mTG-crosslinked Gel–SH composites. **(A)** Small-amplitude oscillatory rheology (1 Hz, 1% strain, 37 °C) shows no significant difference in storage modulus between dense and alginate-porous gels (2.02 ± 0.21 kPa vs. 1.95 ± 0.18 kPa; n = 3; p > 0.05). Loss moduli remain low and unaffected. **(B)** Swelling ratios at 24 h are comparable (6.8 ± 0.5 porous vs. 6.6 ± 0.4 dense; p > 0.05). **(C)** Optical transmittance (350–700 nm) through 1.8 mm disks confirms >60% T at 405 nm; repeated photo-activation causes <3% coumarin photobleaching. **(D)** Live/Dead confocal images (Day 7) show ≥93% viability across conditions, including light-irradiated samples; ΔT at gel surface <1 °C during illumination. MTT assays showed no metabolic differences between light and dark groups. Imaging: upper row 4×, lower row 20×. Scale bars – 100 µm & 50 µm.

Rheological measurements showed that the addition and subsequent dissolution of alginate porogen did not significantly alter the gel stiffness. The storage modulus (G′) of porous Gel–SH/mTG hydrogels was 2.02 ± 0.21 kPa, comparable to 1.95 ± 0.18 kPa for non-porous controls (p > 0.05; n = 3). The gels remained optically clear and mechanically stable for the 21-day culture period, with no detectable degradation or swelling differences between groups. Throughout the 21-day culture, the mTG-crosslinked Gel–SH network retained its transparency and elasticity. Swelling ratios (6.8 ± 0.5 for porous vs. 6.6 ± 0.4 for dense gels) were not statistically different (p > 0.05). Optical measurements after repeated light exposure confirmed negligible photobleaching of the coumarin residues (<3% absorbance loss at 405 nm after ten pulses). No macroscopic deformation or fragmentation was observed. Overall, these results were consistent with previosluy reported findings highlightung that photo-cleavable hydrogels have can retain mechanical integrity under repeated light exposure (Francis and DeForest, 2023; Grim et al., 2015). Importantly, repeated light activation did not alter gel stiffness or crosslink density, confirming that 405 nm illumination specifically targeted the photocleavable linker rather than the Gel–SH backbone (Figure 2).

### Photo-triggered BMP-2 release kinetics

**FIGURE 3:**
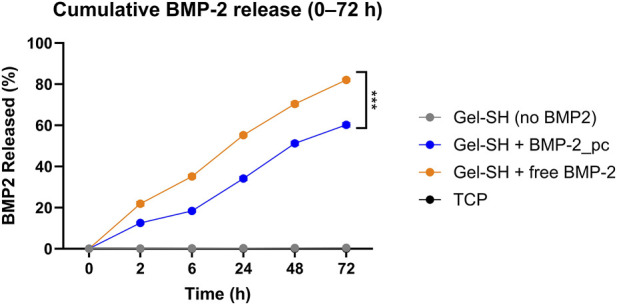
Photocleavage-mediated BMP-2 release. Cumulative BMP-2 release over 72 h from coumarin-tethered scaffolds in dense and porous Gel–SH/mTG formulations under Light (405 nm, 60 s, 25 mW·cm^−2^) versus Dark. Light pulses induce a rapid burst followed by sustained release; dark controls show minimal diffusion.

The photo-responsive marine Gel–SH/mTG system exhibited precise and repeatable BMP-2 liberation upon 405 nm illumination. Spectroscopic analysis confirmed that mTG-crosslinked gels maintained >60% optical transmittance at 405 nm for 1.8 mm-thick disks, ensuring efficient light penetration without scattering artifacts from residual alginate). A single 60-s light pulse produced a burst release of 21.3% ± 2.9% of the immobilized BMP-2 within the first 2 h. Cumulative release increased to 58.7% ± 4.1% by 72 h, whereas non-illuminated controls released <6% of the total load during the same period (p < 0.001) suggesting that BMP-2 retention was dominated by covalent tethering rather than physical adsorption. ELISA-based mass balance analysis confirmed efficient covalent immobilization of BMP-2 within the Gel-SH/mTG network. Apparent loading efficiency approached unity, with values consistently approximating 100% (Table 2), indicating minimal loss during conjugation and gel fabrication. Importantly, the mTG-crosslinked gelatin network remained mechanically stable over the entire culture period, indicating that BMP-2 liberation was governed by photocleavage of the coumarin linker rather than degradation of the hydrogel matrix. Using this approach, the loading efficiency attested at 98,564 ± 0.880% (mean ± SD, n = 3), confirming efficient covalent tethering with minimal loss during processing. Overall, these results are consistent with previous findings highlighting that light-triggered release of BMP-2 in hydrogels, achieved using 405 nm labile linkers (Azagarsamy and Anseth, 2013) and spatiotemporal release can enhance osteogenesis (Barati et al., 2016). Importantly, no premature BMP-2 release was detected during gelation or the overnight equilibration phase, validating the orthogonality of the maleimide–thiol coupling and the enzymatic crosslinking chemistry (Figure 3).

### Oxygen diffusion and mechanical properties of alginate-porous scaffolds

**FIGURE 4:**
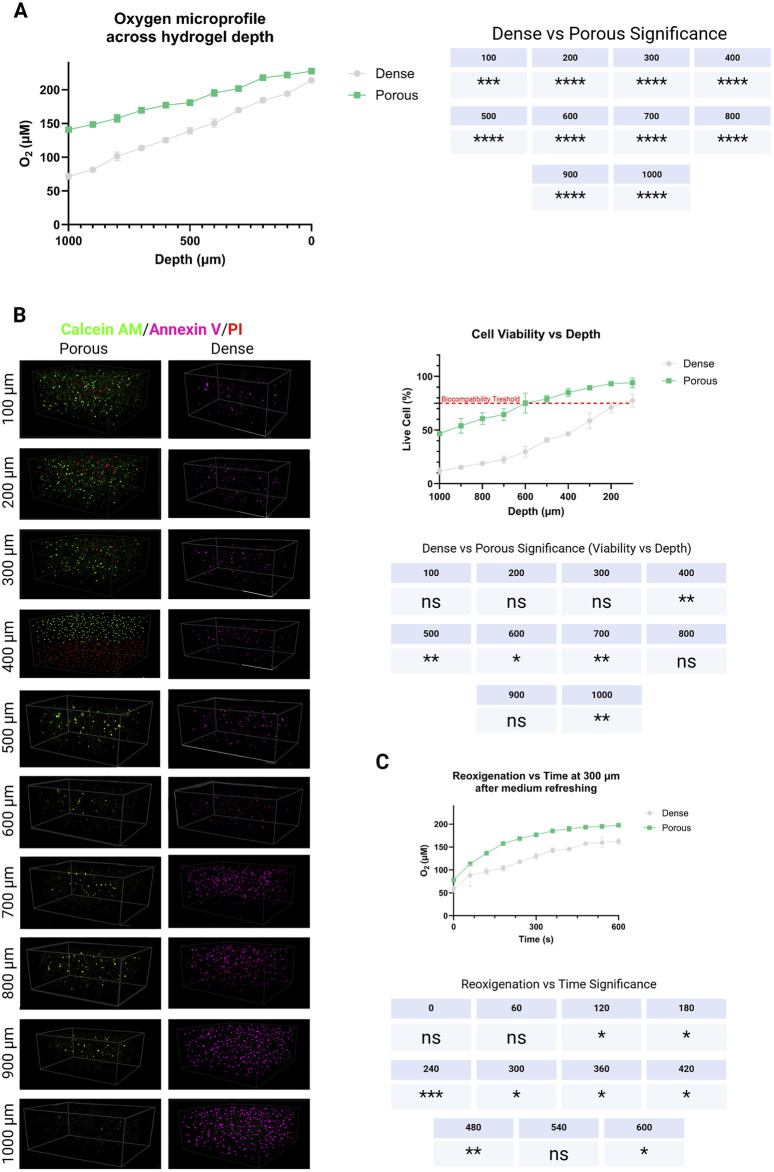
Alginate-generated microporosity improves oxygen transport in cell-laden gels. **(A)** Steady-state O_2_ depth profiles (0–1,000 µm) show higher oxygen across the construct in porous vs. dense gels, with the largest benefit between 200 and 400 µm. **(B)** Z-stacks confocal images at different depth into the hydrogel (100–1,000 µm). Under elevated metabolic demand, the separation between porous and dense increases, indicating superior mass transport in microporous networks, as highlighted by the cell viability quantification. **(C)** Re-oxygenation at 300 µm following medium exchange normalizes within ∼6 min in porous gels but remains slower in dense gels. Mean ± SD, repeated-measures ANOVA across depths **(A–B)** and two-way ANOVA over time × formulation **(C)**; Sidak *post hoc*.

After leaching of the sacrificial alginate, micro-porous channels (25–120 µm) became visible throughout the Gel–SH matrix. Clark-type microelectrode profiling revealed that these channels increased dissolved oxygen concentration at 200–400 µm depth by Δ + 34 ± 7 µM relative to dense, non-porous controls. This finding was consistent with a previous report showing that porous hydrogel architectures can improve mass transport and oxygenation in 3D cell cultures (Kim et al., 2020). During continuous monitoring under standard culture conditions, O_2_ levels returned to equilibrium within approximately 6 min after illumination, indicating efficient diffusion and no persistent oxygen gradients inside the gel (Figure 4).

### Cytocompatibility of encapsulated hDPSC

Encapsulated hDPSC exhibited excellent viability and metabolic stability within the mTG-crosslinked Gel–SH hydrogels. Live/Dead confocal imaging demonstrated homogeneous cell distribution with minimal aggregation across the construct depth. Viability was ≥93% on Day 7 in all formulations, including light-irradiated samples. The 405 nm light pulses did not induce photothermal damage, as temperature changes at the gel surface remained <1 °C during exposure (Figures 2D, 4B). Quantitative MTT assays corroborated the qualitative observations, showing steady metabolic activity at Day 7 with no significant difference between illuminated and dark conditions (p > 0.05). These data confirmed that both the mTG enzymatic crosslinking and the photo-triggering protocol were fully compatible with sensitive dental stem cells. Morphologically, hDPSC retained rounded, within microcavities generated by alginate leaching, however they did not compacted into spheroids, showing an initiation of spindle-like networking. Moreover, to evaluate whether coumarin photoproducts generated during visible-light photoactivation exert adverse cellular effects, DPSC were exposed to conditioned media collected from illuminated BMP-2–free hydrogels. This approach decoupleed potential photoproduct toxicity from BMP-2–mediated biological activity (Lee et al., 2017). Cell viability analysis revealed no significant differences between cells cultured in CM_light and CM_dark, with viability consistently exceeding 95% and comparable to untreated controls (Supplementary Figure S3). In contrast, the positive cytotoxicity control induced a marked reduction in viable cells, confirming assay sensitivity. ROS measurements using the DCFDA assay showed no increase in intracellular oxidative stress following exposure to CM_light relative to CM_dark (Supplementary Figure S3C). Fluorescence levels remained at baseline values, whereas hydrogen peroxide treatment resulted in a robust elevation of ROS, validating the assay response. Genotoxicity was assessed via γH2AX immunofluorescence to detect DNA double-strand break signaling. Representative images demonstrated sparse γH2AX foci in both CM_dark and CM_light conditions, similar to untreated controls (Supplementary Figure S3D). Quantitative analysis confirmed no statistically significant increase in the percentage of γH2AX-positive nuclei following exposure to CM_light (Supplementary Figure S3E). In contrast, UV exposure produced a pronounced increase in γH2AX nuclear staining. To assess a worst-case exposure scenario, conditioned media generated using multiple consecutive light pulses were also tested and similarly showed no effect on viability or ROS generation (Supplementary Figure S3F). Together, these results demonstrate that coumarin photoproducts produced under the applied illumination conditions are cytocompatible and do not induce oxidative or genotoxic stress in DPSC.

### Photo-induced osteogenic differentiation

**FIGURE 5:**
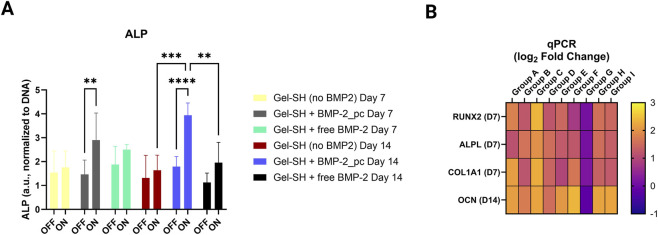
Light-activated BMP-2_pc yields higher osteogenesis. **(A)** ALP activity at Day 7 and Day 14 (normalized to DNA) shows significant increases with daily 405 nm pulses, maximized in Group C. **(B)** qPCR heatmap: early upregulation of RUNX2, ALPL, COL1A1 at Day 7 and late OCN induction at Day 14 under photo-triggered conditions. Statistics: two-way ANOVA (factors = Light, Formulation) with Sidak *post hoc*; mean ± SD.

The biofunctional response of hDPSC depended strongly on both BMP-2 tethering and light activation. After 7 days of osteogenic culture, ALP activity in the light-stimulated Gel–SH + BMP-2_pc group (Group C) was 2.3-fold higher than in the dark-kept counterpart (p < 0.01) and 1.8-fold higher than in gels containing equivalent free BMP-2 without photocontrol (Group E, F, p < 0.01). These enhancements persisted at Day 14, indicating that repeated daily uncaging pulses maintained a sustained osteogenic stimulus (Figure 5).

Gene-expression profiling supported these enzymatic findings. qPCR analysis showed marked upregulation of early and late osteogenic markers in the light-triggered BMP-2_pc group: RUNX2 2.6×, ALPL 2.1×, and COL1A1 1.7× at Day 7, followed by OCN 3.1× at Day 14 compared with free BMP-2 controls (p < 0.05 for all comparisons). In contrast, groups lacking BMP-2 or kept in darkness exhibited only basal expression levels, similar to 2D TCP controls. These results confirm that the photocleavable BMP-2 system effectively translated optical input into transcriptional activation of osteogenesis-related genes, in accordance with previous findings showing that GelMA-based BMP-2 delivery scaffolds enhance osteogenic differentiation in hMSCs (Yuan et al., 2025).

### Matrix mineralization

**FIGURE 6:**
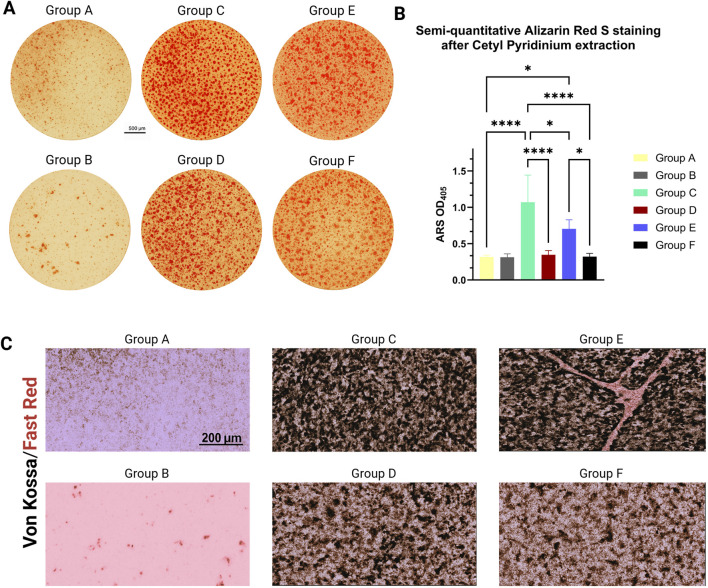
Mineralization (Day 21). **(A)** Representative Alizarin Red S micrographs for the various experimental groups. Identical imaging settings; scale bars as indicated. **(B)** Quantified ARS (OD405) shows maximal mineral deposition in Light-activated BMP-2_pc constructs, exceeding both dark controls and free BMP-2 (two-way ANOVA with Sidak *post hoc*; mean ± SD). N/A, ARS imaging provides qualitative spatial information, whereas extracted OD405 values represent total mineral content normalized to DNA. **(C)** Von Kossa staining from the various groups confirms mineral phase localization (scale bar 200 µm.

By Day 21, mineral deposition was visibly increased in light-activated BMP-2_pc constructs (Figure 6). Alizarin Red S staining produced dense, homogeneous red matrices with minimal background. Quantitative extraction revealed a 2.7-fold increase in calcium-bound dye relative to non-illuminated BMP-2_pc gels and a 3.4-fold increase versus Gel–SH-only controls (p < 0.001). Free BMP-2 formulations showed intermediate mineral levels, suggesting partial protein diffusion and lower local concentration at the cell–matrix interface. Also, even though most uncaged BMP-2 is liberated early, the hydrogel group achieves equal or higher late mineralization than continuous soluble BMP-2, supporting the hypothesis that early instructed commitment and local presentation can sustain downstream osteogenic programs. Von Kossa/nuclear fast red staining further confirmed these results. Overall, these findings demonstrate that photo-triggered BMP-2 release promotes localized and sustained matrix mineralization within the encapsulating hydrogel, validating the spatiotemporal design principle of the material, and showing accordance with previous findings, though in nanoclays, highlighting how sustained retention of active BMP-2 drives robust mineralisation (Kim et al., 2020).

### BMP signalling blockade

**FIGURE 7:**
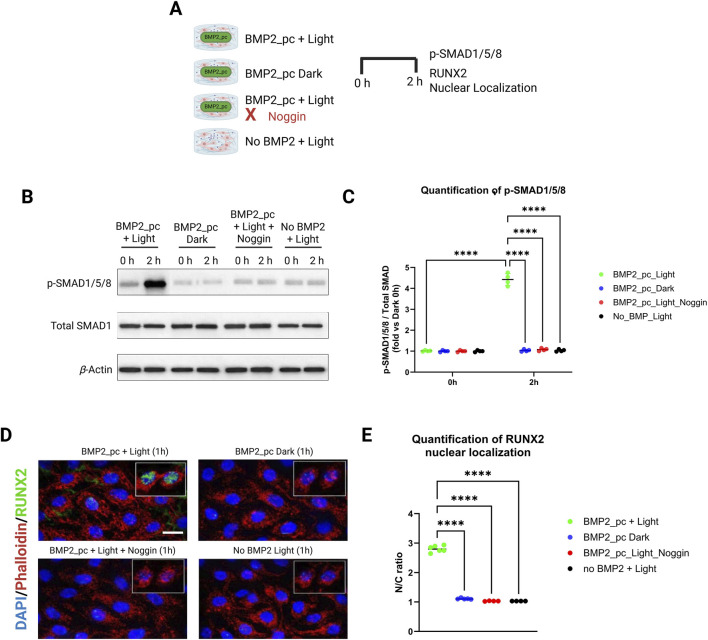
Light-triggered BMP-2 uncaging activates canonical BMP signaling and RUNX2 nuclear translocation in hDPSC and is abolished by BMP pathway blockade. **(A)** Experimental timeline: Gel–SH/mTG hydrogels containing tethered BMP-2_pc were exposed to a single 405 nm pulse (25 mW·cm^−2^, 60 s; 1.5 J·cm^−2^). hDPSC were harvested at 0 and 2 h post-pulse. Noggin (500 ng·mL^−1^) was applied 45 min prior to illumination to block BMP signaling. **(B)** Representative immunoblot showing rapid phosphorylation of SMAD1/5/8 following photoactivation in BMP-2_pc + Light samples, with minimal activation in Dark or No BMP + Light controls. BMP blockade abolishes p-SMAD induction. **(C)** Densitometric quantification of p-SMAD1/5/8 normalized to total SMAD (fold-change vs. dark control). **(D)** Confocal immunofluorescence demonstrates RUNX2 nuclear enrichment at 1 h after photoactivation in BMP-2_pc + Light, which is absent under BMP blockade and in dark/light-only controls (DAPI, nuclei). **(E)** Quantification of RUNX2 nuclear localization (N/C ratio).

To directly test whether photoactivation of the hydrogel induces canonical BMP signaling, early intracellular events downstream of BMP receptor engagement were examined (Figure 7A). A single 405 nm light pulse applied to BMP-2_pc–containing hydrogels induced a rapid and transient increase in SMAD1/5/8 phosphorylation in encapsulated hDPSC. p-SMAD1/5/8 levels increased markedly by 2 h (Figures 7B,C) (Kopf et al., 2012). In contrast, dark-kept BMP-2_pc hydrogels and light-exposed BMP-free Gel–SH controls exhibited only basal p-SMAD levels, indicating that illumination alone did not activate BMP signaling. Importantly, pre-treatment with the BMP antagonist Noggin completely abolished light-induced SMAD1/5/8 phosphorylation, confirming that pathway activation was specifically mediated by BMP-2 signaling rather than nonspecific photobiological effects. Activation of SMAD signaling translated also into functional transcriptional responses (Ahnfelt-Rønne et al., 2010; Zhang et al., 2024). Immunofluorescence analysis revealed pronounced RUNX2 nuclear translocation in hDPSC within 1 h following light activation of BMP-2_pc hydrogels (Figure 7D). Quantitative analysis showed a significant increase in the RUNX2 nuclear/cytoplasmic ratio in the BMP-2_pc + Light group compared to dark controls and BMP-free light-exposed gels (p < 0.01; Figure 7E) (Phimphilai et al., 2006; Jang et al., 2012). This nuclear accumulation was absent in the presence of Noggin, demonstrating that RUNX2 activation depended on BMP receptor engagement (Ahnfelt-Rønne et al., 2010; Zhang et al., 2024).

### ROS assessment following photoactivation

To exclude phototoxic effects associated with repeated 405 nm illumination, intracellular reactive oxygen species (ROS) levels were assessed in encapsulated hDPSC using the DCFDA assay. As shown in Supplementary Figure S4, a single light pulse (25 mW·cm^−2^, 60 s; radiant exposure 1.5 J·cm^−2^) did not induce a measurable increase in ROS compared with dark controls. Quantitative fluorescence analysis confirmed comparable DCFDA intensities between illuminated and non-illuminated samples, whereas treatment with hydrogen peroxide (positive control) produced a robust ROS signal (Eruslanov and Kursmartsev, 2010). Importantly, no delayed ROS accumulation was observed in the hours following photoactivation, indicating that the applied illumination regimen is not associated with acute or latent oxidative stress in encapsulated hDPSC (Hopkins et al., 2016; Zein et al., 2018).

Together, these data establish a direct mechanistic sequence linking light-triggered BMP-2 uncaging to canonical SMAD activation and early osteogenic transcriptional regulation.

## Discussion

This study demonstrates a visible-light–programmable morphogen delivery system capable of releasing bioactive BMP-2 in a temporally controlled manner within a fully cytocompatible, marine-derived hydrogel matrix. By combining thiolated fish gelatin (Gel–SH) (Xiang and Cui, 2021), enzymatic crosslinking via microbial transglutaminase (mTG), and a 405 nm-cleavable coumarin photocage (de Gracia Lux et al., 2015), we created a platform that enables precise, non-destructive control of osteogenic signaling in encapsulated human dental pulp stem cells (hDPSC). The system achieves spatiotemporal modulation of BMP-2 bioavailability without relying on diffusion-based release or photoinitiated polymerization, both of which can compromise protein integrity and cell viability.

The approach integrates three distinct principles: orthogonal light chemistry for on-demand biochemical activation (Ruskowitz and DeForest, 2018; 32; Bojtár et al., 2020; Gupta et al., 2025; Neumann et al., 2023) enzyme-driven crosslinking for maintaining biofunctionality (Chen et al., 2005; Sood et al., 2022), and marine biomaterial engineering for ecological and physiological compatibility (Saeki et al., 2020; Lin et al., 2023). Together, these components yield a biohybrid scaffold that mirrors the dynamic nature of native tissue environments, where biochemical signals are transient and locally modulated rather than constant.

Conventional BMP-2 delivery methods - adsorption, covalent immobilization, or bulk encapsulation - often suffer from uncontrolled burst release, rapid depletion, and low spatial precision (Kempen et al., 2008; Hettiaratchi et al., 2020). In contrast, the coumarin photocage strategy allows predictable cleavage under visible light, translating optical input into biochemical output with reproducible kinetics (Qi et al., 2024; Grim et al., 2015). Each 405 nm pulse released approximately one-fifth of the residual BMP-2 load, enabling graded and repeatable dosing over multiple days. This modular control mirrors the pulsatile signaling characteristic of natural osteogenesis, where cells respond to intermittent morphogen exposure rather than continuous stimulation (Qi et al., 2024; Yi et al., 2020). BMP-2 release in the present system is decoupled from hydrogel degradation and instead governed by orthogonal photochemical cleavage, enabling precise temporal control over growth-factor availability while maintaining matrix integrity.

An important consideration for visible-light–responsive biomaterials is the potential generation of oxidative stress during photoactivation. Although temperature increases were negligible under the applied illumination conditions, photochemical side effects such as ROS generation could still compromise cell function (Zein et al., 2018). Our results demonstrate that 405 nm photoactivation at a radiant exposure of 1.5 J·cm^−2^ does not induce intracellular ROS accumulation in hDPSC (Supplementary Figure S4), supporting the photochemical safety of the selected stimulation parameters. This finding is consistent with previous reports indicating that short-duration blue-light exposures within this energy window are well tolerated by mammalian cells and do not elicit phototoxic responses (Hopkins et al., 2016; Rapp and DeForest, 2020). Together, these data reinforce the suitability of the applied light regimen for repeated, on-demand activation of BMP-2 release in cell-laden hydrogels.

Morevoer, photocleavable linkers are powerful tools for spatiotemporal control of bioactive signaling; however, their translational application requires rigorous evaluation of photoproduct safety (Weinstain et al., 2020). Coumarin-based photocages, while widely used in visible-light–responsive biomaterials, generate low-molecular-weight photoproducts upon cleavage that must be demonstrated to be biologically inert under relevant exposure conditions.

The absence of ROS generation following exposure to CM_light is particularly relevant, as blue-violet light has been associated with oxidative stress at higher radiant exposures or prolonged illumination times (Lee et al., 2017). In our system, the combination of moderate irradiance, short exposure duration, and high optical transparency of the hydrogel matrix appears to avoid photochemical side effects. These findings are consistent with previous reports indicating that coumarin photolysis products generated under visible-light conditions are well tolerated by mammalian cells when appropriate light doses are used (López-Corrales et al., 2023).

Unlike diffusion-based systems, the covalent tethering of BMP-2_pc to Gel–SH ensured that the protein remained spatially localized until photoactivated (Qi et al., 2024), minimizing off-target effects and reducing the risk of ectopic mineralization - a known clinical limitation of uncontrolled BMP-2 release (James et al., 2016). Importantly, because the gelation chemistry (mTG crosslinking) was orthogonal to the photocleavable linker, the protein remained stable and inactive until intentionally triggered, overcoming the long-standing challenge of temporal selectivity in growth-factor delivery (Contessi Negrini et al., 2021).

Also, the work here proposed raises a question concerning whether the relatively rapid liberation of BMP-2 following photoactivation is compatible with the sustained osteogenic differentiation and late-stage mineralization observed at later time points. Classical osteogenic protocols frequently rely on continuous supplementation of soluble BMP-2 over several weeks to maintain differentiation. However, the evidence here highlighted indicates that BMP-2 might function also as an instructional morphogen, where early, temporally confined signaling events can commit progenitor cells to the osteogenic lineage, with downstream differentiation programs persisting even after extracellular ligand levels decline. To directly interrogate this distinction, the photo-programmed hydrogel system was benchmarked against two conventional reference conditions: continuous soluble BMP-2 supplementation and transient (time-dosed) soluble BMP-2 exposure in TCP cultures. These controls allowed direct comparison between sustained, transient, and matrix-localized BMP-2 signaling. Notably, photo-triggered BMP-2 delivery induced osteogenic gene expression, ALP activity, and late-stage mineralization that were comparable to or exceeded those achieved with continuous soluble BMP-2 exposure, despite a substantially shorter extracellular BMP-2 availability window (Halloran et al., 2020; Wang et al., 2014; Sagner and Briscoe, 2017; Chhabra et al., 2019).

This apparent decoupling between ligand persistence and phenotypic outcome can be rationalized by several complementary mechanisms. First, the photo-cleavage strategy produces high local concentrations of BMP-2 at the cell–matrix interface, a presentation mode fundamentally distinct from diluted soluble delivery and known to enhance receptor clustering and downstream SMAD signaling. Second, early activation of master osteogenic regulators such as RUNX2 initiates transcriptional cascades that become self-sustaining and no longer require continuous ligand input. Third, the remaining tethered BMP-2 fraction, together with autocrine and paracrine BMP signaling from differentiating cells, likely contributes to reinforcement of the osteogenic program (Samorezov et al., 2016; Hauff et al., 2015).

Importantly, comparison with the pulsed soluble BMP-2 control suggests that temporal patterning itself is a critical determinant of osteogenic outcome, rather than total BMP-2 exposure time alone (Beederman et al., 2013). The ability to deliver BMP-2 in user-defined, discrete pulses may therefore better mimic developmental morphogen dynamics than continuous dosing and offers a strategy to reduce total growth-factor usage while preserving efficacy (Beederman et al., 2013; Halloran et al., 2020).

Taken together, these findings support a paradigm in which early, spatially localized, and temporally patterned BMP-2 signaling is sufficient to drive sustained osteogenic differentiation, challenging the assumption that prolonged extracellular growth-factor presence is strictly required (James et al., 2016; Zara et al., 2011). Within this framework, photo-programmable delivery systems provide a unique opportunity to decouple biochemical instruction from material degradation and to explore new dosing designs in regenerative medicine.

Another central requirement for optically programmable biomaterials is demonstration of causal coupling between light input and biological output. While sustained osteogenic differentiation provides functional validation, it does not alone prove pathway specificity. Here, the system presented tries to also close this causal loop by showing that photoactivation of BMP-2_pc hydrogels induces rapid, BMP-dependent intracellular signaling events in hDPSC.

The observed SMAD1/5/8 phosphorylation within 2 h following illumination is consistent with canonical BMP receptor activation kinetics and confirms that uncaged BMP-2 remains biologically competent to engage its receptors (Phimphilai et al., 2006; Liao et al., 2026). The transient nature of this activation mirrors physiological morphogen signaling, where short-lived ligand exposure initiates durable transcriptional programs rather than continuous stimulation (Zinski et al., 2018).

Crucially, both SMAD phosphorylation and RUNX2 nuclear translocation were suppressed by Noggin, demonstrating that these effects are not attributable to light exposure, matrix mechanics, or nonspecific photostimulation (Phimphilai et al., 2006). This blockade experiment establishes a direct cause-effect relationship: light-triggered BMP-2 release is necessary and sufficient to activate osteogenic signaling.

RUNX2 nuclear accumulation represented another key commitment step in osteogenic differentiation, acting downstream of SMAD signaling to regulate osteoblast-specific gene expression (Phimphilai et al., 2006; Javed et al., 2008). Its rapid translocation following photoactivation provides a mechanistic explanation for the enhanced ALP activity, osteogenic gene expression, and matrix mineralization observed at later time points. Importantly, these early signaling events occur well before measurable extracellular matrix deposition, supporting the concept that temporally confined BMP-2 signaling can irreversibly program lineage commitment.

These findings also address concerns regarding the apparent mismatch between early BMP-2 release kinetics and late-stage osteogenic outcomes. Rather than requiring continuous exposure, the data support a model in which discrete, photo-timed BMP-2 pulses initiate transcriptional cascades that persist beyond ligand availability, consistent with developmental morphogen dynamics and prior reports on pulsatile BMP signaling (Zinski et al., 2018).

Moreover, the substitution of traditional porcine or bovine gelatin with marine-derived Gel–SH introduced both ethical and functional advantages (Ma et al., 2021). Fish gelatin contains a higher proportion of low-melting-point amino acids and fewer hydrophobic residues, resulting in increased hydration and lower gel stiffness at equivalent concentrations (Liu et al., 2020; Sharifi et al., 2021). When enzymatically crosslinked by mTG, however, the resulting network achieved mechanical properties (G′ ≈ 2 kPa) comparable to mammalian analogues (Buscaglia et al., 2022; Liu et al., 2020) while remaining fully transparent to 405 nm light. Transparency was critical for efficient photoactivation through the construct thickness, ensuring homogeneous release within the encapsulated cell volume (Sharifi et al., 2021).

Beyond optics, the marine origin of Gel–SH aligns with a bio-circular design ethos, minimizing animal-waste dependence and valorizing oceanic biomaterials - a conceptual continuation of those paradigms that merge biotechnology with sustainable marine sourcing (Zhang et al., 2019). The gelatin backbone provided natural integrin-binding motifs (Arg–Gly–Asp), which supported excellent hDPSC attachment, spreading, and viability throughout the culture period, confirming that biochemical compatibility was preserved despite thiolation and enzymatic crosslinking (Yoon et al., 2016).

Using microbial transglutaminase instead of photoinitiated polymerization addressed two recurring limitations of visible-light biofabrication: premature uncaging of light-sensitive components and potential cytotoxicity of radical initiators (Yang et al., 2018; Yamada et al., 2024). The enzymatic route offered mild, oxygen-independent crosslinking under physiological conditions (37 °C, pH 7.4) (Buscaglia et al., 2022), maintaining high cell viability (>90%) and protein activity. The resulting network remained stable for 21 days without degradation or leaching of residual enzyme, similarly to previous reports (Gupta et al., 2021). Rheological stability (G′ ≈ 2 kPa) and swelling ratios (∼6.7) indicated an equilibrium consistent with previous reports for mTG-gelled fish gelatin (Buscaglia et al., 2022; Long et al., 2017), confirming that the material maintained mechanical consistency even under repeated illumination cycles.

Importantly, the enzymatic approach allowed for separate tuning of chemical and mechanical cues: BMP-2 coupling was governed by thiol-maleimide chemistry, while crosslinking density was determined enzymatically (Yoo et al., 2021; Gilchrist et al., 2021). This modularity will facilitate future integration of other bioactive molecules (e.g., VEGF, FGF2) using orthogonal linkers without re-optimizing the gelation process (Molina et al., 2025).

The incorporation of sacrificial alginate microdomains significantly improved the local microenvironment for encapsulated cells by increasing internal oxygen diffusion (Augustine et al., 2023). Following leaching, the porous Gel–SH network exhibited a mean Δ[O_2_] of +34 ± 7 µM compared to dense controls, confirming efficient mass transport without structural compromise. Oxygen availability is crucial in three-dimensional stem-cell cultures, particularly during early osteogenic differentiation, where hypoxic conditions can inhibit matrix mineralization and cell metabolism, pushing through chondrogenesis rather than osteogenesis. The improved diffusion likely contributed to the uniform viability and differentiation observed across the gel depth.

Encapsulated hDPSC responded robustly to the photo-triggered release of BMP-2. The daily light pulses generated reproducible biochemical activation, reflected by significant increases in ALP activity and osteogenic gene expression relative to both dark controls and free-BMP-2 groups, consistently with previous reports (Liao et al., 2020). The magnitude of RUNX2, ALPL, and OCN upregulation (2–3×) matched or exceeded those reported for static BMP-2 delivery systems, demonstrating that temporal stimulation may be more effective than sustained exposure in driving lineage commitment (Ingwersen et al., 2022).

By Day 21, light-activated constructs exhibited nearly threefold higher mineral deposition compared with unilluminated equivalents. This result emphasizes the functional relevance of programmable release (Kim et al., 2020) - allowing the same total BMP-2 dose to produce stronger osteogenic outcomes when presented in discrete, photo-timed pulses (Jansen et al., 2010). This phenomenon parallels physiological morphogen waves observed during development and suggests that periodic biochemical signaling may better synchronize transcriptional and metabolic responses in DPSC (Wei et al., 2020).

Previous photoresponsive scaffolds often relied on ultraviolet irradiation or free-radical photodegradation, posing cytotoxic risks and limited penetration depth (Moon et al., 2023). The present system utilizes visible light at 405 nm, which lies within a clinically relevant optical window, minimizing DNA damage and enabling straightforward delivery using inexpensive LEDs or fiber optics (Rapp and DeForest, 2023). Unlike prior methacrylated GelMA formulations, where the same wavelength is used for polymerization, the mTG-crosslinked Gel–SH network allows the photonic trigger to be dedicated solely to controlled release, eliminating unwanted activation during fabrication (Yu et al., 2020).

This decoupling represents a critical step toward real clinical deployment. It enables chairside or intraoperative photoactivation without compromising the structural integrity of the scaffold. Moreover, because instances of the coumarin cage be adapted for red-shifted variants (520–630 nm) exist (Kaufmann et al., 2023), future iterations could achieve even deeper tissue penetration using minimally invasive light delivery via micro-LED catheters (Weinstain et al., 2020).

Despite its promising results, several limitations remain. First, light penetration at 405 nm is restricted to approximately 2–3 mm in hydrated tissue; therefore, thicker constructs or *in vivo* applications will require either higher power, embedded light guides, or the development of red-shifted photocages to reach clinically relevant depths (Lim et al., 2019). Second, while the enzymatic crosslinking is biocompatible, residual mTG activity may persist *in vivo* and should be carefully evaluated for long-term implantation to exclude immune or fibrotic responses (Yang et al., 2016). Third, although the use of fish-derived gelatin enhances sustainability, batch variability in amino-acid composition and thiol substitution could affect mechanical reproducibility; standardized sourcing or recombinant marine collagen analogues may be required for regulatory translation (Rýglová et al., 2023).

From a biological standpoint, the study was conducted only *in vitro* using encapsulated hDPSC, which, although an established osteogenic model, does not replicate the vascularization and mechanical dynamics of bone defects, as already established by our group (Rýglová et al., 2023). Future studies should include co-culture with endothelial cells and validation in a rat calvarial defect model to assess bone integration, angiogenesis, and immune compatibility (Shanbhag et al., 2021). Long-term biodegradation profiles and potential inflammatory responses to the released coumarin fragments must also be examined.

Future development will focus on refining multi-wavelength programmable hydrogels, where different photocages release distinct biofactors - e.g., sequential activation of VEGF and BMP-2 (Azagarsamy and Anseth, 2013) - to mimic the temporal hierarchy of bone healing. Incorporating fiber-optic or micro-LED arrays within the hydrogel bulk could permit localized photostimulation at greater depths, while integration with optoelectronic sensing systems would enable real-time feedback on mechanical or biochemical cues (Rapp and DeForest, 2023).

Additionally, leveraging the marine biomaterial framework opens pathways for sustainable scaling and customization. Marine collagen and polysaccharides can be blended to tailor stiffness, degradation rate, and optical clarity. These biomaterials are naturally abundant and align with the growing need for environmentally responsible medical materials (Zhang et al., 2019).

## Conclusion

This work presents a novel class of enzymatically crosslinked, marine-derived, light-responsive hydrogels designed to achieve precise, non-destructive control over osteogenic signaling. By integrating thiolated fish gelatin (Gel–SH) with microbial transglutaminase (mTG) crosslinking and a 405 nm-cleavable coumarin photocage (Yang et al., 2016; Yung et al., 2007), we established a platform that converts visible-light energy into programmable biochemical stimulation for encapsulated human dental pulp stem cells (hDPSC). The system demonstrates that photo-triggered release of BMP-2 can effectively surpass traditional static or diffusion-based delivery methods, offering enhanced osteogenic differentiation, sustained enzymatic activity, and significant matrix mineralization - all while maintaining high cytocompatibility and mechanical stability (Zhu et al., 2020). The use of marine gelatin introduces both ecological and functional advantages, enabling optical transparency, renewable sourcing, and native bioactivity within a sustainable biomaterial framework (Yoon et al., 2016). The approach represents a blue biotech strategy where marine polymers and photonics intersect to guide regeneration with temporal precision (Francis and DeForest, 2023). Importantly, the enzymatic gelation process eliminates the need for photoinitiators or radicals, ensuring complete spectral separation between scaffold formation and light-controlled release. This dual orthogonality - chemical and optical - ensures predictable, repeatable, and safe activation of morphogen release within clinically relevant light windows. Overall, the combination of marine Gel–SH, mTG crosslinking, and visible-light photochemistry establishes a foundation for next-generation, light-controlled regenerative materials. These findings highlight temporal patterning as an emerging design variable in tissue engineering, suggesting that regenerative therapies of the future may rely not only on biochemical composition but also on the timing of biochemical exposure. In this sense, the study advances the concept of “dosing with photons” - a paradigm in which clinicians could 1 day activate, modulate, or terminate regenerative cues *in situ* through harmless bursts of visible light, achieving precise, adaptive control over tissue repair processes.

## Data Availability

The original contributions presented in the study are publicly available. This data can be found here: https://zenodo.org/records/18279506.
