# Correction: Visible-light–triggered BMP-2 release from enzymatically crosslinked marine collagen–alginate hydrogel blends enhances osteogenesis in dental pulp stem cells

**DOI:** 10.3389/fphys.2026.1855975

**Published:** 2026-05-06

**Authors:** Francesco Torelli

**Affiliations:** 1 SOSD Odontostomatologia Chirurgica e Speciale, Ospedale Riuniti delle Marche, Ancona, Italy; 2 Microfluidic Innovation Centre, Paris, France

**Keywords:** alginate porogen, BMP-2, coumarin photocage, dental pulp stem cells, enzymatic crosslinking, light-triggered release, marine collagen, osteogenesis

Affiliation “SOSD Odontostomatologia Chirurgica e Speciale, Ospedale Riuniti delle Marche, Italy” was omitted for author Francesco Torelli. This affiliation has now been added for author Francesco Torelli.

Author Francesco Torelli was erroneously assigned to affiliation “Tissue Engineering Group, Translational Oral Research Unit, Department of Clinical Dentistry, University of Bergen, Bergen, Norway”. This affiliation has now been removed for author Francesco Torelli.

In the **Acknowledgements**, Shuntaro Yamada is listed as “Shuntaro Yamada (University) without any further specification. It should read “Shuntaro Yamada (University of Bergen).

The original version of this article has been updated.

