# Supplementary material for: Visible-light–triggered BMP-2 release from enzymatically crosslinked marine collagen–alginate hydrogel blends enhances osteogenesis in dental pulp stem cells
Source: Front Physiol. 2026 Feb 17;17:1743209. doi: 10.3389/fphys.2026.1743209 (PMC12953112; doi:10.3389/fphys.2026.1743209)
Supplement: Supplementary file 1 [file DataSheet2.docx]

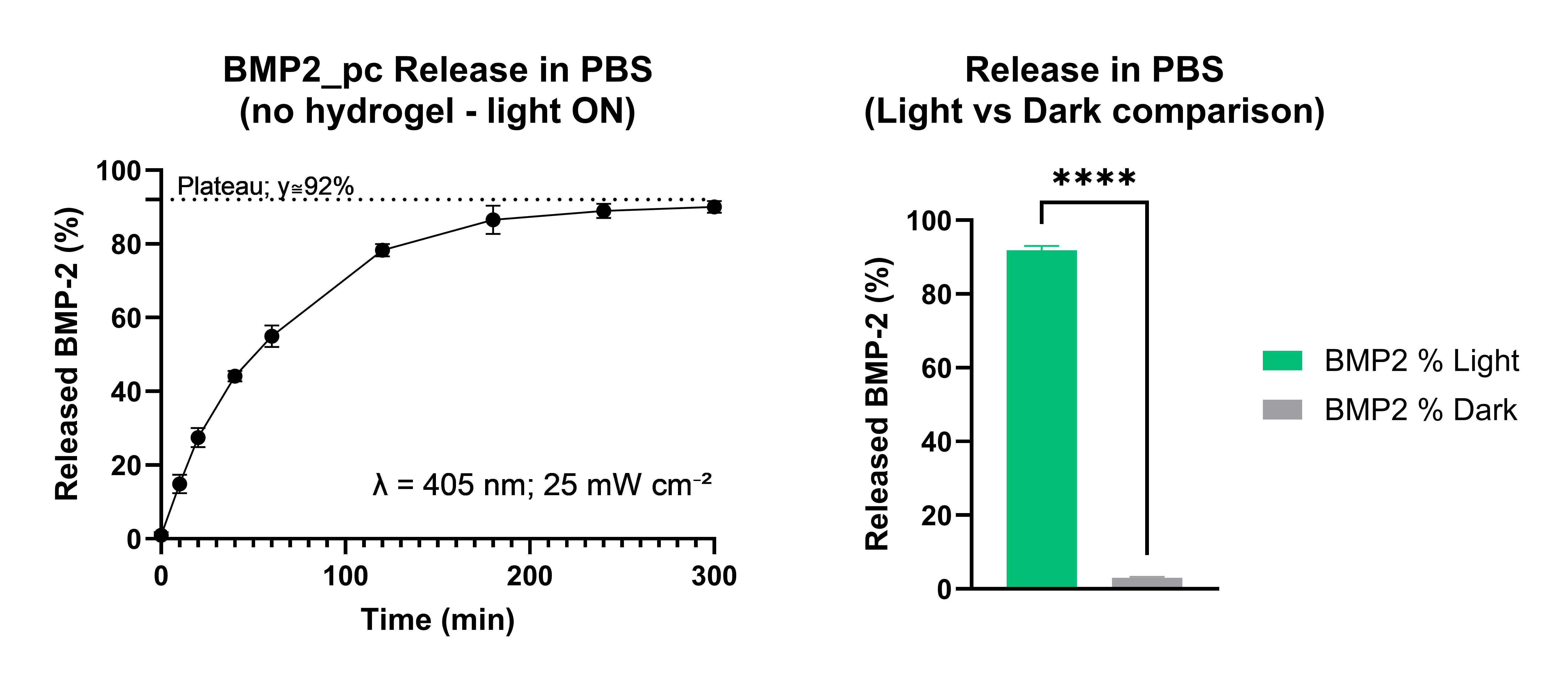

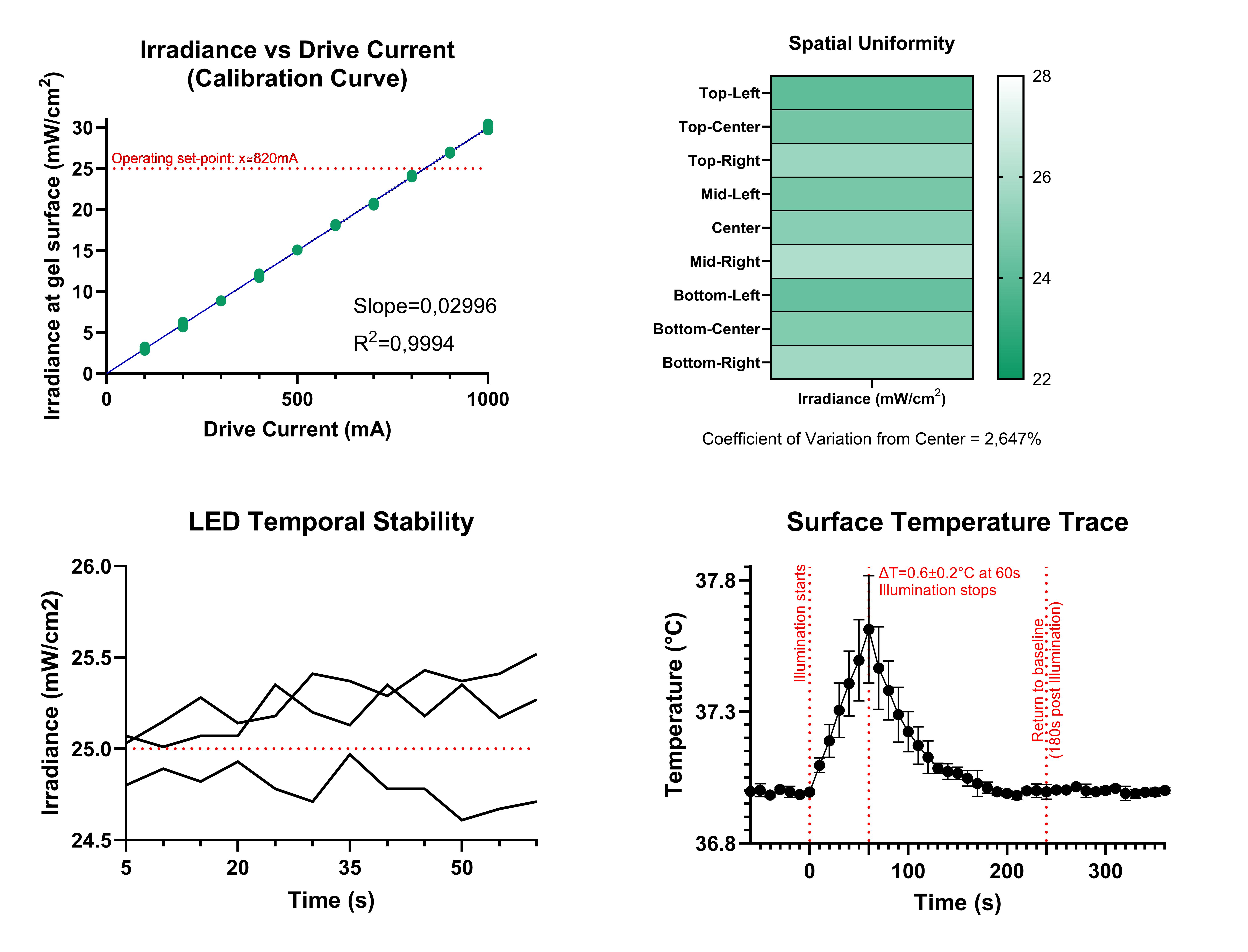

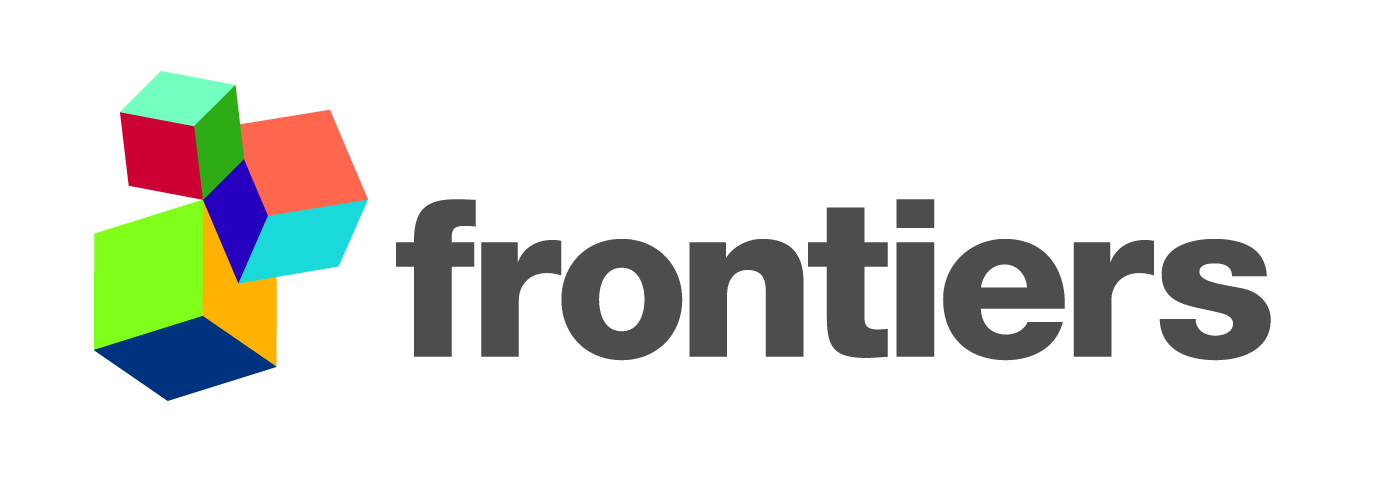
Supplementary Material

## Supplementary Figures

##
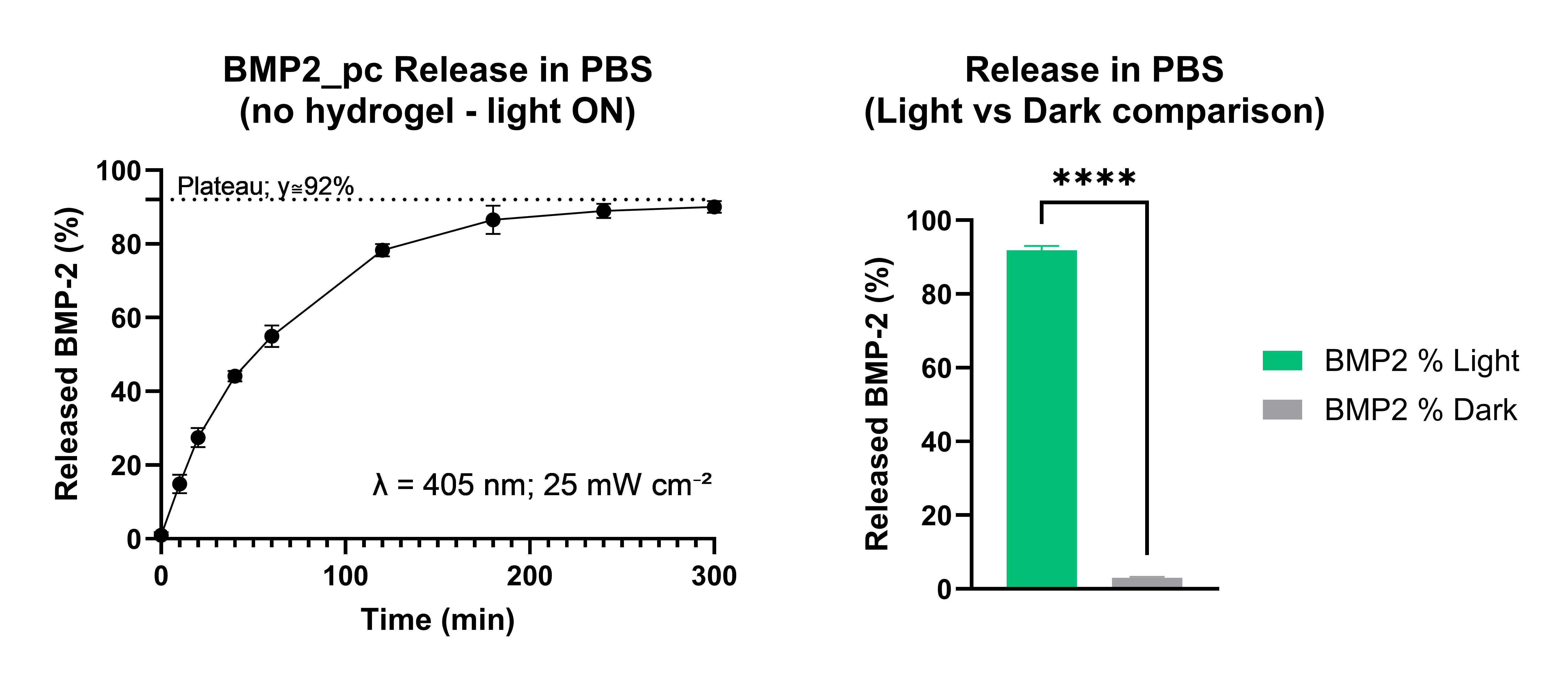


## Supplementary Figure 1. Photocage cleavage kinetics of BMP-2_pc under blue light*.* (Left) Released BMP-2 (%) vs illumination time showing exponential release. (Right) Comparison of total BMP-2 release after 300 min under light vs dark control (p < 0.001). Data shown as mean ± SD (n = 3).


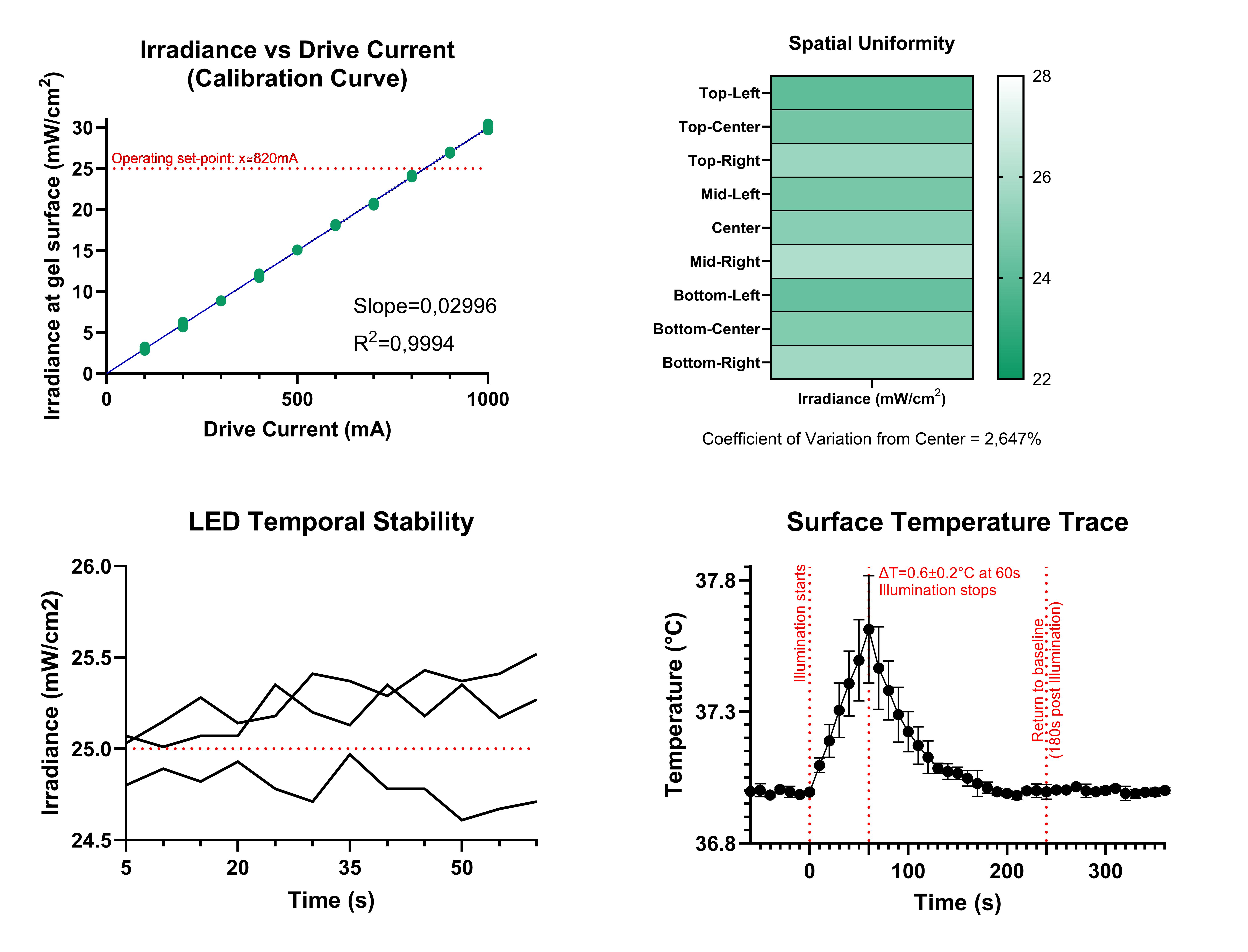


**Supplementary Figure** 2**.** **LED Calibration and Uniformity.** (A) Irradiance–current calibration of the 405 nm LED system showing linear response and experimental set-point (25 mW cm⁻²). (B) Spatial irradiance mapping over the gel plane, confirming ± 2.647 % coefficient of variation. (C) Temporal stability trace over 60 s indicating drift < 2.5 %. (D) Surface temperature during 60 s exposure showing ΔT < 1 °C. All data represent mean ± SD (n = 3 independent calibrations).


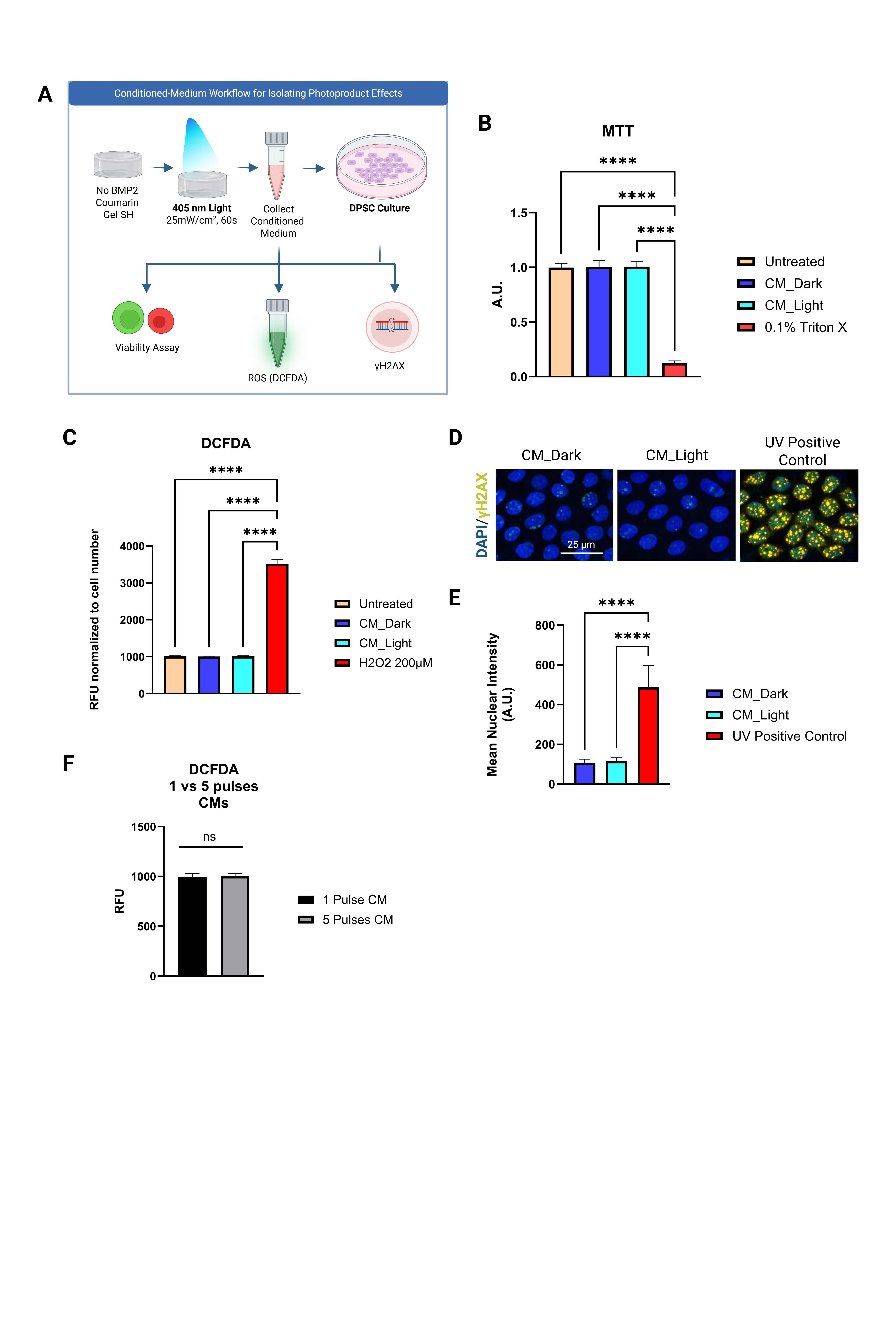


**Figure S3. Cytocompatibility and genotoxicity assessment of coumarin photoproducts generated during photoactivation.** (A) Schematic of conditioned-medium (CM) workflow to isolate coumarin photoproduct effects from BMP-2 signaling. BMP-2-free coumarin-functional gels were exposed to 405 nm light (25 mW·cm⁻², 60 s; radiant exposure 1.5 J·cm⁻²), incubated in medium to generate CM, and CM was applied to DPSC cultures for downstream assays. (B) Cell viability by MTT exposure to CM_dark or CM_light shows no cytotoxicity relative to untreated controls. (C) DCFDA assay indicates no increase in ROS in CM_light compared to CM_dark; H₂O₂ serves as a positive control. (D) Representative immunofluorescence images of γH2AX (DNA damage marker) and DAPI. (E) Quantification of γH2AX-positive nuclei shows no evidence of increased DNA damage in CM_light; UV exposure used as positive control. (F) “Worst-case” multi-pulse CM exposure does not alter ROS and viability compared to single-pulse CM. Data shown as mean ± SD. Statistics: one-way ANOVA with Tukey post hoc (α = 0.05). Scale bar: 25 µm.


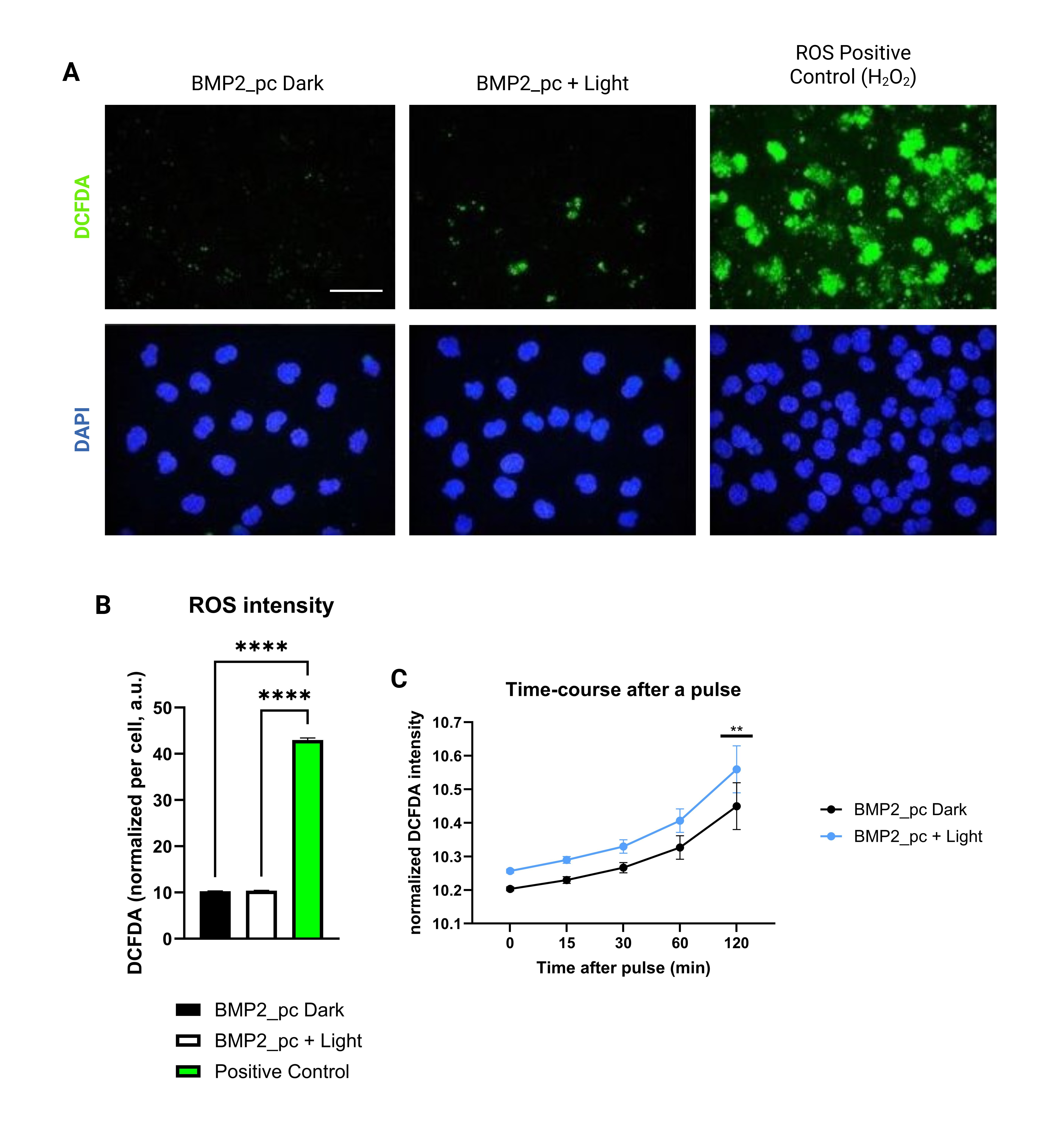


**Supplementary Figure 4**. **405 nm photocaging activation does not increase intracellular ROS in encapsulated hDPSC.** (A) Representative DCFDA fluorescence images of hDPSC in hydrogels kept in the dark, exposed to a single 405 nm pulse (25 mW·cm⁻², 60 s; 1.5 J·cm⁻²), or treated with H₂O₂ as a positive ROS control. (B) Quantification of normalized DCFDA fluorescence per cell shows no significant difference between dark and light conditions, while the positive control exhibits a robust ROS increase. (C) Time-course analysis after illumination confirms no delayed ROS elevation following photoactivation. Data are mean ± SD; n = 3 independent constructs; statistics by one-way ANOVA with Tukey post hoc.
