# Supplementary material for: Visible-light–triggered BMP-2 release from enzymatically crosslinked marine collagen–alginate hydrogel blends enhances osteogenesis in dental pulp stem cells
Source: Front Physiol. 2026 Feb 17;17:1743209. doi: 10.3389/fphys.2026.1743209 (PMC12953112; doi:10.3389/fphys.2026.1743209)
Supplement: Supplementary file 2 [file DataSheet1.docx]

Supplementary Material

## Supplementary Figures

##
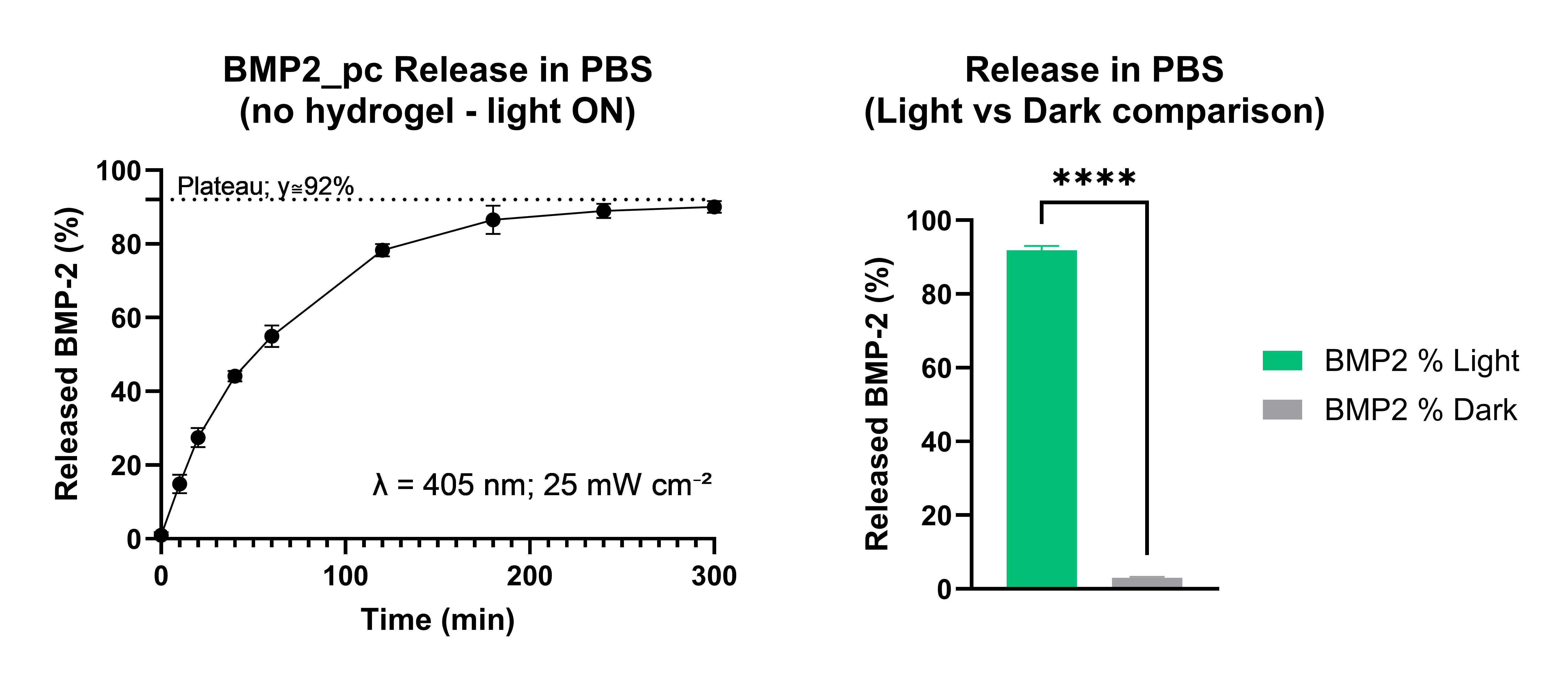


## **Supplementary Figure 1.** **Photocage cleavage kinetics of BMP-2_pc under blue light***.* (Left) Released BMP-2 (%) vs illumination time showing exponential release. (Right) Comparison of total BMP-2 release after 300 min under light vs dark control (p < 0.001). Data shown as mean ± SD (n = 3).


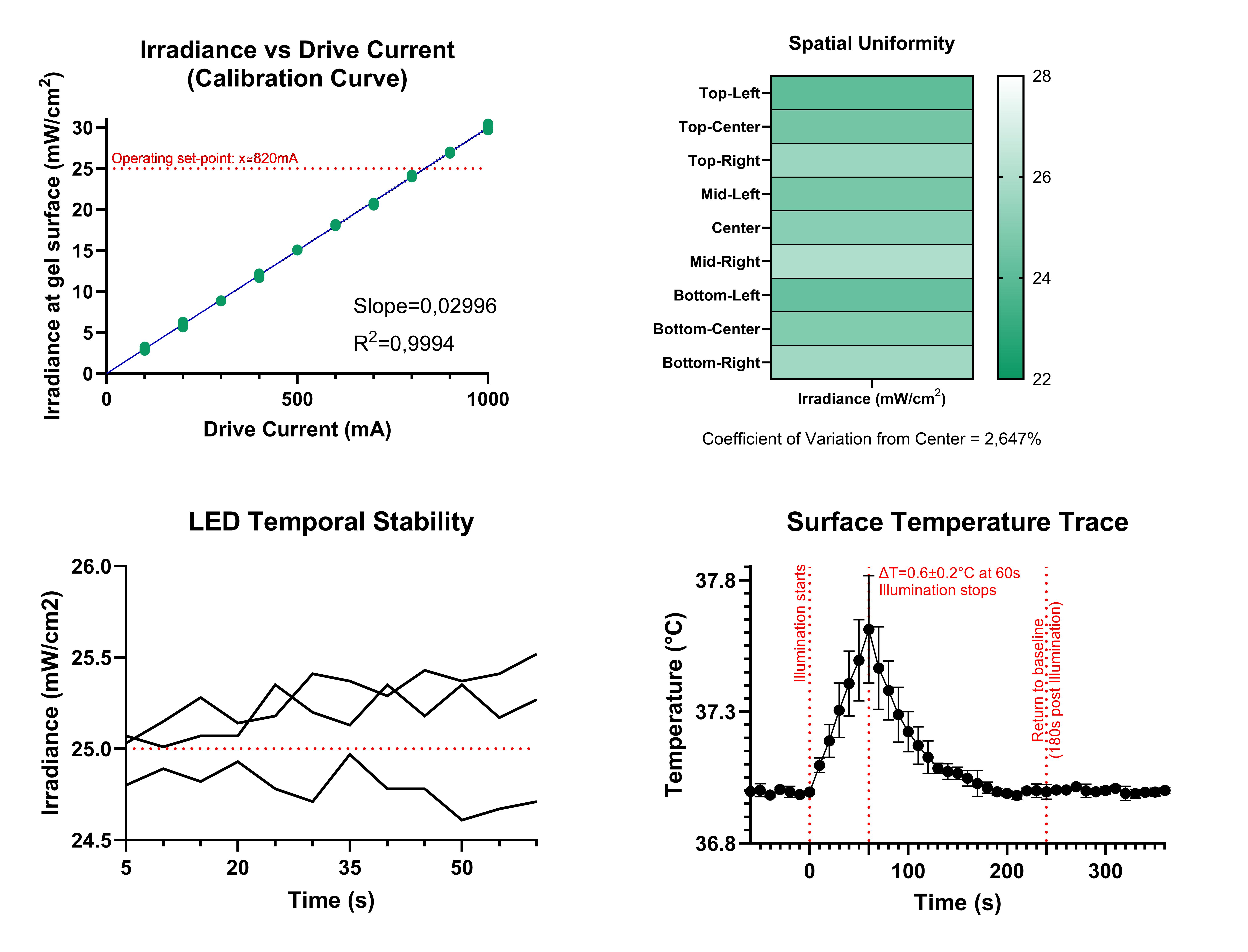


**Supplementary Figure 2.** **LED Calibration and Uniformity.** (A) Irradiance–current calibration of the 405 nm LED system showing linear response and experimental set-point (25 mW cm⁻²). (B) Spatial irradiance mapping over the gel plane, confirming ± 2.647 % coefficient of variation. (C) Temporal stability trace over 60 s indicating drift < 2.5 %. (D) Surface temperature during 60 s exposure showing ΔT < 1 °C. All data represent mean ± SD (n = 3 independent calibrations).
